# Protein dynamic communities from elastic network models align closely to the communities defined by molecular dynamics

**DOI:** 10.1371/journal.pone.0199225

**Published:** 2018-06-20

**Authors:** Sambit Kumar Mishra, Robert L. Jernigan

**Affiliations:** 1 Bioinformatics and Computational Biology Program, Iowa State University, Ames, Iowa, United States of America; 2 Roy J. Carver Department of Biochemistry, Biophysics and Molecular Biology, Iowa State University, Ames, Iowa, United States of America; University of Michigan, UNITED STATES

## Abstract

Dynamic communities in proteins comprise the cohesive structural units that individually exhibit rigid body motions. These can correspond to structural domains, but are usually smaller parts that move with respect to one another in a protein’s internal motions, key to its functional dynamics. Previous studies emphasized their importance to understand the nature of ligand-induced allosteric regulation. These studies reported that mutations to key community residues can hinder transmission of allosteric signals among the communities. Usually molecular dynamic (MD) simulations (~ 100 ns or longer) have been used to identify the communities—a demanding task for larger proteins. In the present study, we propose that dynamic communities obtained from MD simulations can also be obtained alternatively with simpler models–the elastic network models (ENMs). To verify this premise, we compare the specific communities obtained from MD and ENMs for 44 proteins. We evaluate the correspondence in communities from the two methods and compute the extent of agreement in the dynamic cross-correlation data used for community detection. Our study reveals a strong correspondence between the communities from MD and ENM and also good agreement for the residue cross-correlations. Importantly, we observe that the dynamic communities from MD can be closely reproduced with ENMs. With ENMs, we also compare the community structures of stable and unstable mutant forms of T4 Lysozyme with its wild-type. We find that communities for unstable mutants show substantially poorer agreement with the wild-type communities than do stable mutants, suggesting such ENM-based community structures can serve as a means to rapidly identify deleterious mutants.

## Introduction

The dynamic nature of globular proteins allows them to sample multiple conformations around their native equilibrium conformation. Such intrinsic dynamics is conferred by their geometry and can be influenced by events such as ligand binding or even binding of a partner enzyme [1]. Such events typically shift the conformational equilibrium of proteins allowing them to sample new conformations by lowering energy barriers, which were not accessible from the native state [2,3]. Such dynamic plasticity is characteristic for protein function [4–6]. It facilitates signal transduction through allosteric regulation as well as allowing bio-molecular machines to undergo large scale conformational changes from their native state essential for their function [7–9].

Inspecting the conformational ensemble arising due to the dynamic nature of proteins gives immediate insight into how different parts of a protein move with respect to one another. Some regions may exhibit highly correlated motions while others may be anti-correlated in their motions. A map describing the extent of inter-residue dynamical correlation between residues can then be used to create a graphical representation which portrays the dynamic nature of a protein [10]. In such a graph, the nodes represent the residues and the edges are weighted by the dynamical correlation for a residue pair. Residue blocks which are highly correlated in their motions and move as a cohesive unit can then be identified from these graphs and are commonly referred to as dynamic communities [11,12]. These communities may correspond to structural domains in proteins; however, they are often smaller modules whose motions relate to the protein’s function.

Previous studies have used both normal mode analysis (NMA) and molecular dynamics (MD) approaches to detect structural domains and dynamic communities in proteins. Hinsen *et al*. [13] used normal modes to compute residue-level deformation energy and then, identified dynamically rigid segments using a threshold based on the deformation energy. Kundu and co-workers [14] used Gaussian Network Model (GNM) [15] to partition protein structures into domains using the eigenvector corresponding the lowest non-zero eigenvalue, also referred to as the Fiedler vector. In another study, Yesylevskyy *et al*. [16] used GNM to obtain a correlation matrix describing inter-residue dynamics and used it to calculate a “correlation matrix of correlation patterns” which essentially describes the overlap between the correlation patterns for different residues. Then they performed hierarchical clustering on this matrix to obtain rigid communities. A similar study used correlations in residue dynamics calculated from normal mode analysis to decompose protein kinases into residue blocks that are dynamically cohesive [17].

Other studies where MD simulations were used to identify the rigid domains have also been carried out. Potestio *et al*. [18] used MD simulations to obtain conformational ensemble describing the essential dynamics of proteins and then used dominant eigenvectors from covariance matrix describing the variation in the ensemble to identify rigid domains. McClendon and co-workers [10] performed a thorough investigation of protein kinase A using microsecond-scale MD simulations and then identified communities using inter-residue dynamical correlations from the trajectory with the Girvan-Newman clustering scheme to understand the mechanism of allostery in the enzyme. A similar study on Bruton’s tyrosine kinase by Chopra *et al*. [19] revealed that inspecting the community changes for the enzyme’s mutant form reveals the changes in the allosteric coupling in the enzyme. In another study, Yao and co-workers [20] performed community analysis on G proteins using 80-ns MD simulations to identify residues playing a critical role in the allosteric coupling between functional domain interfaces.

MD simulations do provide a high resolution dynamic image of a protein describing detailed motions of individual atoms at different time points. However, most proteins require energy minimization with respect to an all-atom potential prior to any simulation, a computationally demanding task for larger structures. Moreover, to observe large-scale conformational changes as often seen in the case of multi-domain proteins, simulations need to be performed on the microsecond to millisecond time-scales, which also require considerable computing power. In such cases, coarse-grained approaches like ENM have an upper hand [15,21,22]. These models adopt a coarse-grained representation for proteins by representing each residue by only its alpha carbon (C^α^). They also implement a simplified potential that uses Hookean springs to connect residue pairs within a cutoff distance to calculate the native state dynamics for proteins. In assuming that the crystal structure of a protein corresponds to a local minimum on the energy landscape and considering it as the native state conformation, these models eliminate the necessity for energy minimization. Owing to their reduced nature, these models require minimal computational resources even for large macromolecular structures. Previous studies have shown that theoretical B-factors calculated using ENM correspond well to the experimental temperature factors [15,21,22]. A study by Leioatts *et al*. suggests that these models provide consistent outcomes irrespective of the details of their formulations and thus, do not strongly depend on their underlying parameters [23]. In addition, normal modes from ENMs show significant overlaps with principal components from both experimental sets of structures as well as with MD ensembles [24] and tuning the inter-residue Hookean springs further improves the correspondence with MD [25]. Comparing the dynamics between ENM and MD also suggests that collective motions obtained with ENM from alternate conformations of a macromolecular complex cannot be reliably obtained using multiple runs of MD simulations [26]. Besides, when supplemented with MD, ENMs have also found their applications for generating conformers along transition pathways [27].

In this study, we have performed a large set of comparisons between the dynamic communities obtained from GNM [15] (a type of ENM) and from MD for a set of 44 non-redundant proteins. After applying a systematic hierarchical clustering scheme on the dynamic cross-correlation matrices, we observe a close correspondence between the communities from GNM and MD for specific community levels, characterized by a significantly high value of Cohen’s kappa coefficient [28]. Centrality measures for the weighted dynamic network from GNM and MD also reveal a strong correlation for the closeness centrality values. We also verify the extent of agreement for the inter-residue cross-correlations between GNM and MD by investigating the overlaps of the principal eigenvectors calculated from the dynamic cross-correlation matrices and observe a good overlap. A further analysis of the effect of mutations on communities derived using GNM for T4 lysozyme confirms that highly deleterious point mutations significantly alter the community structure when compared to the neutral mutations. The results from our study open up new avenues for mining dynamic communities in macromolecular structures with ENM and using their changes to screen for deleterious mutants.

## Results

We perform our study on a set of 44 non-redundant proteins (see S1 Table) taken from the MOlecular Dynamics Extended Library (MODEL) database [29]. Each protein has a minimum simulation time of 100 ns for its MD trajectory. We consider only the positions of the residue alpha-carbon atoms of each protein from the trajectory file and calculate the inter-residue dynamical correlations from the respective MD trajectory (*DCC*_*MD*_) using equation 1. In our procedure we consider only the first frame of the MD trajectory of a given protein as its representative structure to render the protein as a mass-spring system. In such a system, each residue is represented by a point mass (its C^α^ atom) and residue pairs within a given distance cutoff (*r*_*c*_) are connected by hypothetical Hookean springs. Such a model is commonly referred to as an elastic network model. The Gaussian Network Model is a formulation of ENM that assumes residue fluctuations to be isotropic in nature. Details concerning the implementation of GNM are provided in the Materials and Methods section.

We construct GNM for each protein by setting the distance cutoff *r*_*c*_ to 7.5 Å and calculate the inter-residue dynamical correlations (*DCC*_*GNM*_) using a subset of 5, 10, 20, 30 and 50 modes (Eq 5). The choice for *r*_*c*_ was based on a preliminary analysis where we identified the *r*_*c*_ that gave the best overlaps between simulation results from GNM and MD. This is followed by a systematic comparison between the inter-residue dynamical correlations from MD and GNM. Initially, we show how closely the dynamic cross-correlation (DCC) matrices from MD and GNM compare with each other for two randomly selected proteins. Following this, we perform more thorough comparisons using the three metrics described below.

- **Kappa coefficient.** The DCC matrix for a protein describes the extent of correlation between the pairs of its C^α^ atoms. We identify blocks of residues that move cohesively (dynamic communities) by first clustering the DCC matrix hierarchically and then, using a cutoff on the height of the dendrogram obtained to identify the required number of communities (*N*_*c*_). In the present study, we identify 2–10 communities (*N*_*c*_ = 2, 3, 4 …, 10) for a given protein. Agreement between the communities from MD and GNM is then assessed with kappa coefficient [28,30].
- **Network centrality.** We model each protein as a weighted network with the nodes corresponding to residues and edges between pairs of residues weighted by their distance transformed dynamical correlations (Eq 6 and Eq 7). Then, we calculate the residue-level closeness centralities and verify the correlations for the centralities obtained from MD and GNM.
- **Overlap between principal eigenvectors.** To assess how well the correlation matrices obtained from MD and GNM compare for a protein, we also perform singular value decompositions of the matrices and then use root-mean square inner product (RMSIP) to evaluate the extent of overlap between the principal eigenvectors from the two systems.

In the final section of this paper, we use GNM to delineate the community structure of wild-type and mutant forms of T4 Lysozyme and to show that elastic models can capture the difference in community structures for the wild-type and mutant forms.

### DCC maps from MD and GNM

**Fig 1:**
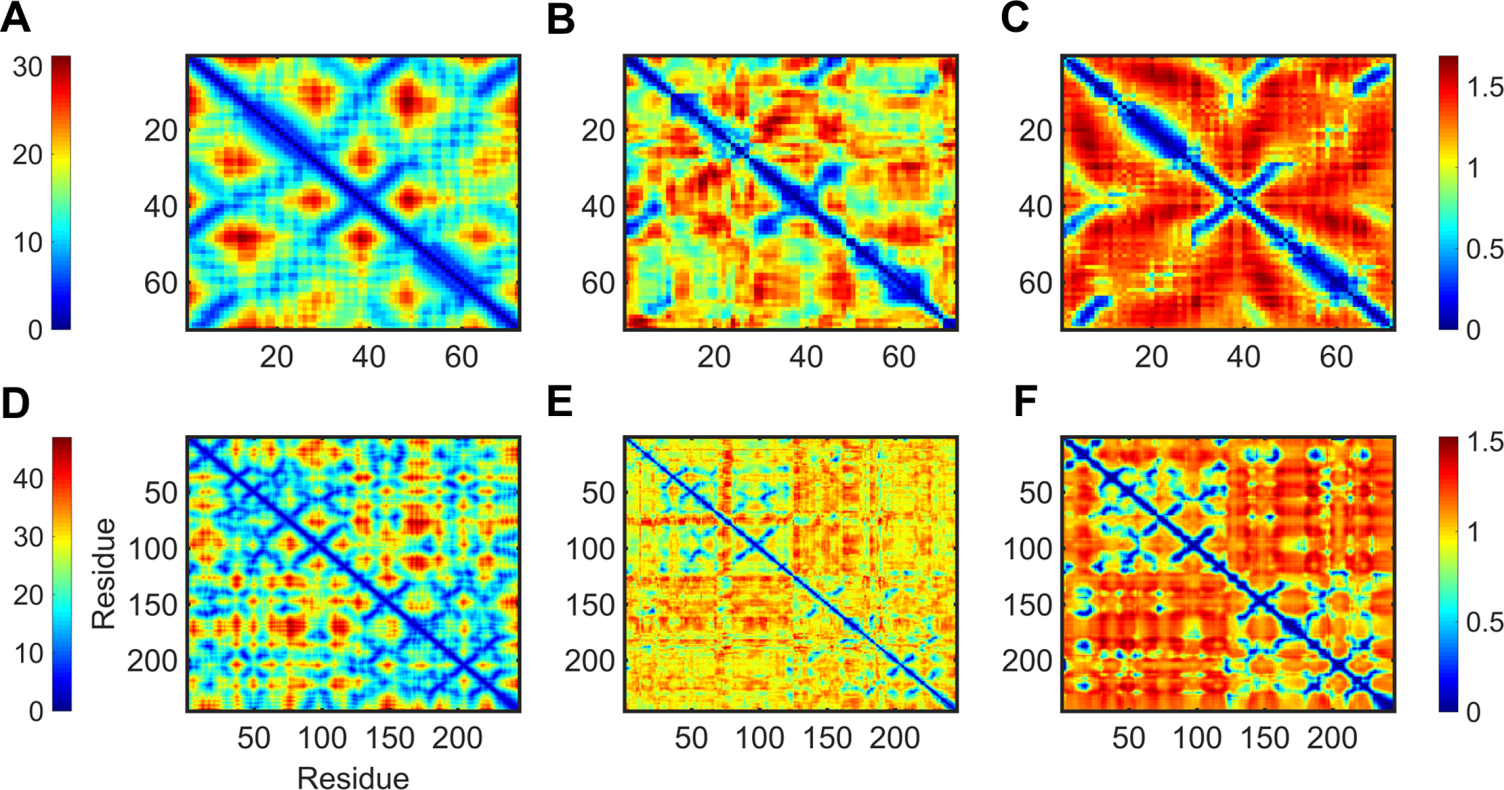
Examples of C^α^-distance maps and distance transformed dynamic cross-correlations from MD and GNM for *i*. **Copper transporter domain from copper transporting ATPase (*top*), and *ii*. alpha-chymotrypsinogen (*bottom*).** For each protein, the figure shows the distance map for alpha-carbons (A and D), *dist_DCC*_*MD*_ (B and E) and *dist_DCC*_*GNM*_ (C and F). The color scale ranges from red (spatially distant regions and least correlated parts) to blue (regions in spatial proximity and most correlated parts). The PDB IDs of the structures used are 1fvq and 1cgi, for *i* and *ii* respectively. For ease of comparison with the C^α^-distance maps, we use *dist_DCC* which has all values on a positive scale rather than *DCC* that has both positive and negative values.

We perform an initial visual inspection of the dynamic maps obtained from MD and GNM to understand the overall extent of agreement for residue correlations from the two methods. Fig 1 describes the dynamic map for two randomly selected proteins from our dataset; *top*: copper transporter domain from copper transporting ATPase (PDB 1fvq), *bottom*: alpha-chymotrypsinogen (PDB 1cgi). The figure shows the distance map between C^α^ atoms (*A*, *D*), distance transformed DCC maps from MD (*B*, *E*) and GNM (*C*, *F*) for the two molecules. We calculated the DCC map for GNM by setting the distance cutoff *r*_*c*_ to 7.5Å and then considering only the 20 non-zero lowest frequency modes as these have often been shown to circumscribe the most energetically favorable conformation fluctuations in proteins [31]. The diagonal elements of the correlation maps describe fluctuations of individual residues while off diagonal elements describe inter-residue correlations or cross-correlations. We note from the outset that there are strong similarities among these representations, corresponding to the secondary structures present in these structures.

The distance map for a protein provides information about the spatial proximity of residues. Spatially close residues are naturally expected to have high correlations in their dynamics. For the two proteins, we observe both MD and GNM showing high inter-residue dynamical correlations for the spatially close residues. However, it is interesting to notice that correlations for residues in spatial proximity are more strongly indicated with the GNM than by MD. The distance transformed cross-correlation and hence, the corresponding cross-correlation maps from MD and GNM exhibit good overall agreement. It is also worth noting that for alpha-chymotrypsinogen, the blocks of residues with high inter-residue dynamical correlation in MD ([1–70], [80–120, 1–70] and [120–220]) are almost closely replicated by GNM. Moreover, the extent of similarity in the correlation profiles of the secondary structure elements (helical regions along the diagonal and anti-parallel beta strands perpendicular to the diagonal) for MD and GNM is quite remarkable.

### Metric based comparisons

#### i. Kappa coefficient

Our objective is to investigate the level of similarity between the communities obtained from MD and GNM. As we identify a range of communities for a protein (*N*_*c*_ = 2, 3, 4 …, 10), we perform a one-to-one comparison between MD and GNM for a given *N*_*c*_. To this end, we first calculate for each protein, the dynamic cross-correlation maps for MD (*DCC*_*MD*_) with Eq 1. Then, we construct GNMs for all proteins using *r*_*c*_ = 7.5 and calculate *DCC*_*GNM*_ using a subset of the low-frequency modes: 5, 10, 20, 30 and 50 modes (Eq 4 and Eq 5). We thus have 5 correlation matrices for a given protein each calculated using a specific subset of modes described above. For a given protein, we then perform hierarchical clustering on the distance transformed *DCC*_*MD*_ (Eq 6) and *DCC*_*GNM*_ (Eq 7) and then truncate the resulting dendrogram to get 2–10 communities. Using kappa coefficient (Eq 8) [28,30], a metric which is used to test inter-rater reliability (extent of agreement between data collectors in assigning same scores to the same variables), we then determine the extent of similarity between the communities from MD and GNM.

**Fig 2:**
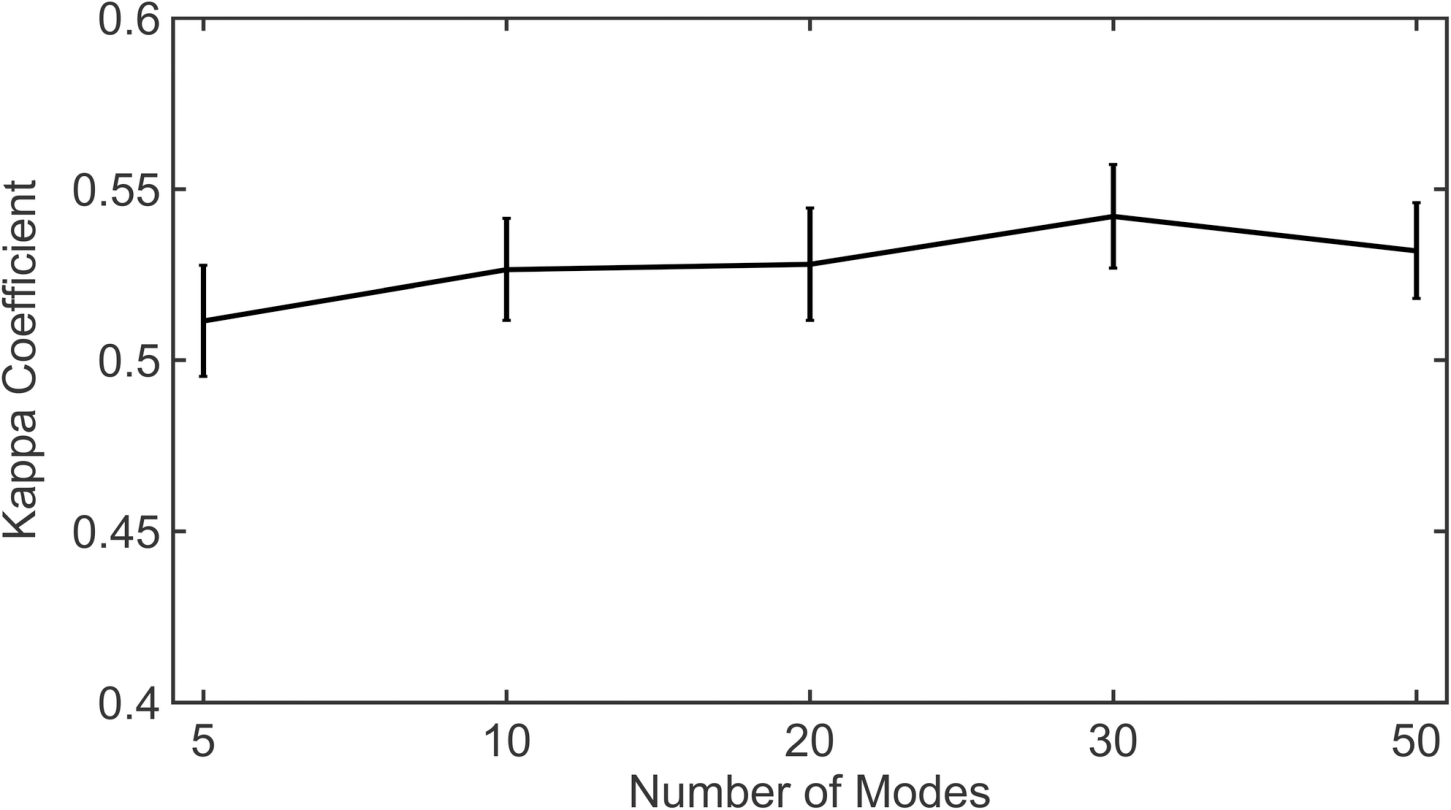
Variation of kappa coefficient with the number of modes. The figure shows the median *Kappa*_*max*_ for all proteins in the dataset for subsets including 5, 10, 20, 30 and 50 modes. Vertical bars represent the standard error of *Kappa*_*max*_.

For a given protein, we consider the *N*_*c*_ (2, 3, 4 … 10) that yields the maximum kappa coefficient (*Kappa*_*max*_) for a chosen subset of modes. For example, if we choose the subset of modes used to calculate *DCC*_*GNM*_ as the first 10, we first calculate the kappa coefficient for all *N*_*c*_ and then choose the particular *N*_*c*_ that gives maximum kappa coefficient and thus, maximum agreement between MD and GNM. Fig 2 shows the median of *Kappa*_*max*_ for each subset of modes used. Similar to correlation coefficients, the kappa coefficient can range from -1 to 1. A value of -1 indicates complete disagreement whereas, 0 indicates the random case. It can be seen that for all subsets of modes used, the median value for *Kappa*_*max*_ is at least 0.5, indicating that the agreement is reasonably good and is not just random. Details of *Kappa*_*max*_ obtained for individual proteins and the respective *N*_*c*_ are provided in S2 Table. We also consider all kappa coefficients for all community levels obtained using the distance cutoff 7.5Å and calculate the median kappa for each subset of modes (S3 Table and S1 Fig). As might be expected, the median kappa when considering all community levels for each subset of modes is smaller than the median of *Kappa*_*max*_ (≈ 0.41). Considering the fact that the conformations sampled by MD might be limited, biased by the trajectory time scale whereas ENMs can sample a relatively broader ensemble independent of time, a kappa coefficient of 0.4 indicates fair agreement between the communities but importantly, the agreement is not random.

**Fig 3:**
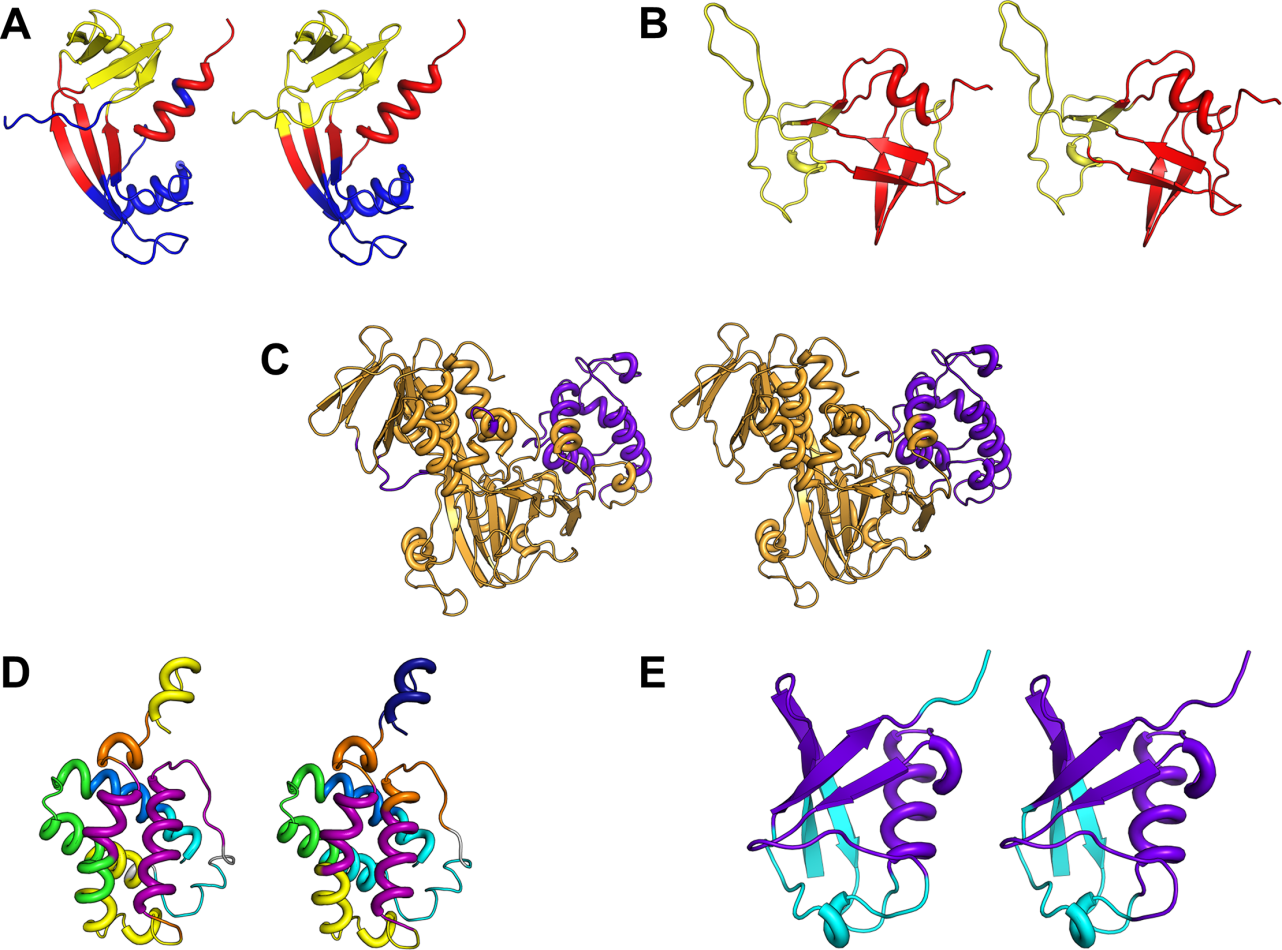
Comparison of communities from MD and GNM. **Mapped communities for five proteins.** (A) Angiogenin (PDB ID: 1agi), (B) Protease (PDB ID: 1nso), (C) Guanine nucleotide dissociation inhibitor (PDB ID: 1gnd), (D) Hemoglobin (PDB ID: 1idr), (E) Ubiquitin (PDB ID: 1ubq). The number of communities (*N*_*c*_) shown for each case corresponds to the case of maximum agreement between MD and GNM given by *Kappa*_*max*_. *DCC*_*GNM*_ calculated with a subset of 20 low-frequency modes was used for each protein to perform calculations for communities.

In Fig 3, we show the communities from MD and GNM mapped onto the structures of 5 proteins (A. Angiogenin, B. Protease, C. Guanine nucleotide dissociation inhibitor, D. Hemoglobin, and E. Ubiquitin). For each protein, the figure shows only the community level *N*_*c*_ that provides the best agreement with MD. The figure clearly depicts the close agreement between the communities from GNM and from MD.

#### ii. Network centrality

The node centrality is computed by modeling a protein as a network where nodes are the C^α^ atoms and the edges are weighted by the correlation in dynamics between a residue pair. Centrality measures tell us the importance of nodes in facilitating the flow of information within the network [32–34]. The most central nodes act as hubs and can be essential to the transmission of information between nodes at the extreme ends of the network. We compare the extent of correlation for residue centralities between GNM and MD.

**Fig 4:**
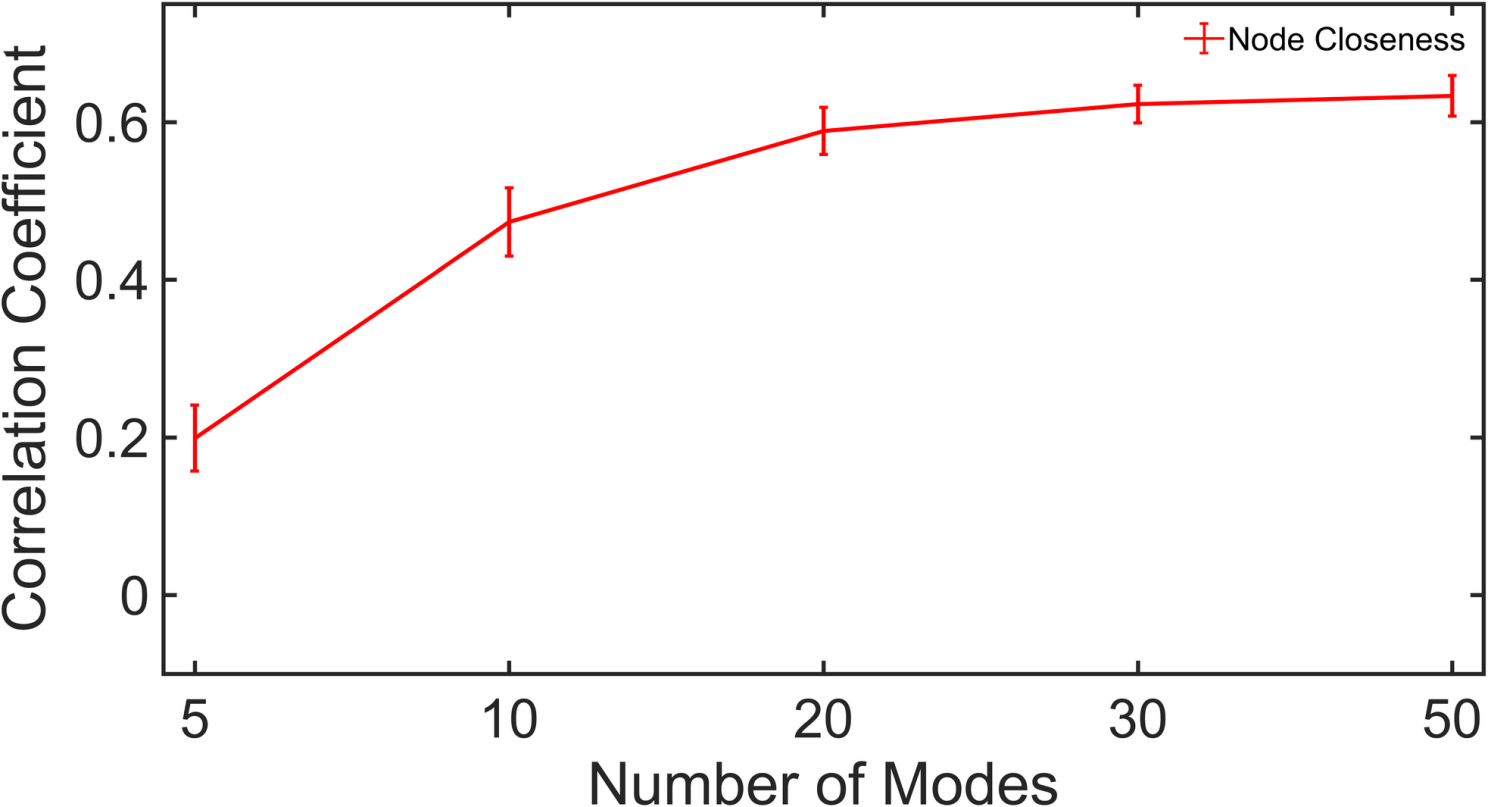
Node centrality correlations. The median correlation for closeness centrality from *DCC*_*GNM*_ with *DCC*_*MD*_ is shown for different subsets of modes for all proteins. Vertical bars give values of standard errors.

We consider the residue closeness centrality, which is the cumulative sum of the lengths of the shortest paths from the residue to all other residues [35,36]. It is also defined as the reciprocal of farness. The centralities calculations were performed using the distance transformed *DCC*_*GNM*_ and *DCC*_*MD*_ (Eq 6 and Eq 7). Fig 4 shows the correlations for the node closeness between MD and GNM where it can be seen that both methods show significantly high correlations in their centralities. It is worth noting that although the maximum correlation is obtained using 50 modes (≈ 0.63), a steep rise in the curve is observed only until 20 modes, after which the curve has almost converged. S4 Table describes the correlations for residue closeness centralities obtained using each subset of modes for individual proteins.

#### iii. Overlap between principal eigen vectors

How well do the dominant motions captured from *DCC*_*GNM*_ quantitatively compare with *DCC*_*MD*_? How many low-frequency GNM modes are required to closely reproduce the correlation pattern from MD? To answer these questions, we investigate the extent of overlap between the principal eigenvectors from *DCC*_*GNM*_ and *DCC*_*MD*_.

**Fig 5:**
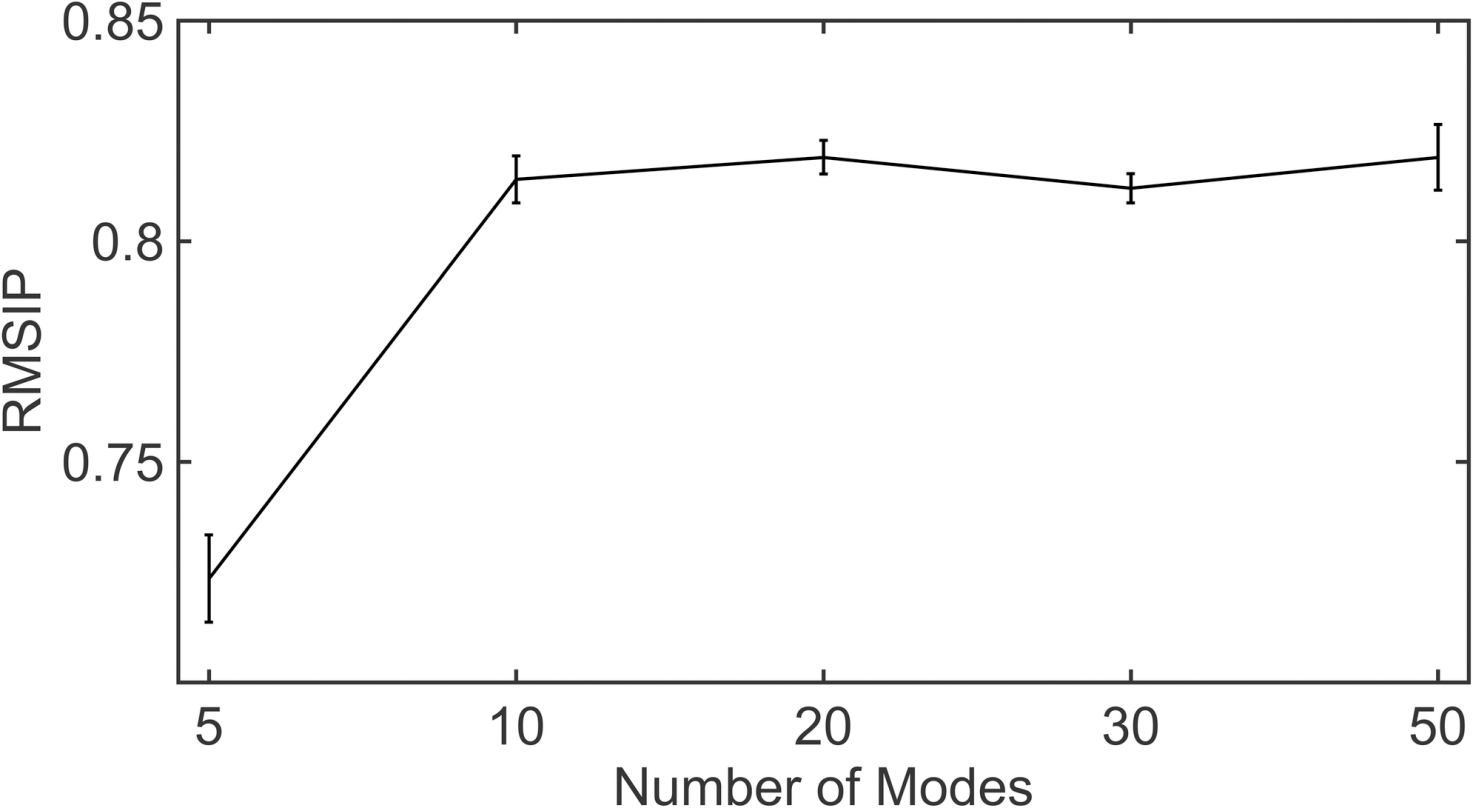
Overlap between principal vectors from *DCC*_*GNM*_ with *DCC*_*MD*_. The figure shows the extent of agreement between the residue cross-correlation matrices from MD and GNM in terms of the principal eigenvectors. The principal eigenvectors are obtained from singular value decomposition of the *DCC*_*GNM*_ and *DCC*_*MD*_ matrices, respectively. The median overlap between the vectors from MD and GNM, computed with RMSIP, is shown for subsets of 5, 10, 20, 30 and 50 modes. Vertical bars represent the standard errors in RMSIP.

Let *U*^*N*^ and *V*^*N*^ be the set of *N* principal eigenvectors obtained upon singular value decomposition (SVD) of *DCC*_*GNM*_ and *DCC*_*MD*_. By principal eigenvectors we are referring to the set of eigenvectors with highest eigenvalues. Because the *DCC* matrix is comparable to a covariance matrix, vectors *U*_*i*_ and *V*_*i*_ are comparable to the principal components of a covariance matrix, capturing the directions of maximum variance from the residue cross-correlation matrix. We inspect the overlap between *U* and *V* using root-mean square inner product (RMSIP) (Eq 9) and quantitatively evaluate the extent of similarity between the two matrices. It is also to be noted that we consider the same number of principal eigenvectors each from *U*^*N*^ and *V*^*N*^ as the subset of modes used. Details about the calculation of RMSIP are provided in Materials and Methods. In Fig 5, we show that the overlaps between the principal eigenvectors of the *DCC*_*GNM*_ and *DCC*_*MD*_ matrices are high. The figure also depicts sharp increases in RMSIP and hence, a steep positive gradient as the subset of modes selected increases from 5 to 10 following which the curve converges. S5 Table gives the RMSIP values of individual proteins for different subsets of low-frequency modes.

### Changes to dynamic communities upon mutations

Mutations can lead to changes in the structure of dynamic communities [19]. We hypothesize that highly unstable mutations tend to change the community structure in a protein more radically than mutations that are less unstable. To test this, we consider 16 mutant structures of T4 Lysozyme crystallized and reported by Mooers *et al* [37]. In their study, the authors investigated the effect of mutating Arg96 on the stability of the enzyme. *ΔΔG* values were reported that indicate changes in the stabilities relative to the wild-type (Table 1). The more negative numbers indicate higher instability. We arbitrarily divide the dataset into two groups: the more unstable mutants (rows 1–8) having *ΔΔGs* between -4.7 and -2.6 and less unstable mutants (rows 9–16), *ΔΔGs* varying between -2.6 and 0. For simplicity, we refer to the more unstable type as *unstable* and the less unstable type as *stable*. We obtain the dynamic communities with GNM using all heavy-atoms from the atomic protein structures and then, with *DCC*_*GNM*_ from 5, 10, 20, 30 and 50 modes, we verify the community agreement for each of the two mutant types with the wild-type with the kappa coefficients.

**Table 1:** Mutants for T4 Lysozyme sorted by *ΔΔG*. The set of PDB structures used to compare the community structure of stable and unstable mutants is given below. The Mutation column gives information on the mutation and has the format “xRy”, where ‘x’ is the residue in the wild-type, ‘y’ the residue in the mutant, and R is the position of mutation in the protein. More negative *ΔΔG* **values** indicate less stable mutant form.

PDB Identifier	Mutation	*ΔΔG* (pH 5.35)	Stability
3c80	R96Y	-4.7000	Unstable
3fi5	R96W	-4.5000	Unstable
3c7z	D89A, R96H	-3.8000	Unstable
3c82	K85A, R96H	-3.6000	Unstable
3c8q	R96D	-3.5000	Unstable
3cdt	R96N	-3.0000	Unstable
3cdv	R96M	-2.7000	Unstable
3c8r	R96G	-2.6000	Unstable
3cdq	R96S	-2.6000	Stable
3c8s	R96E	-2.5000	Stable
3cdo	R96V	-2.4000	Stable
3c7y	R96A	-2.0000	Stable
3c81	K85A	-0.6000	Stable
3c83	D89A	-0.5000	Stable
3cdr	R96Q	-0.3000	Stable
3c7w	R96K	0.0000	Stable
4s0w	None (wild-type)	0	Stable

**Fig 6:**
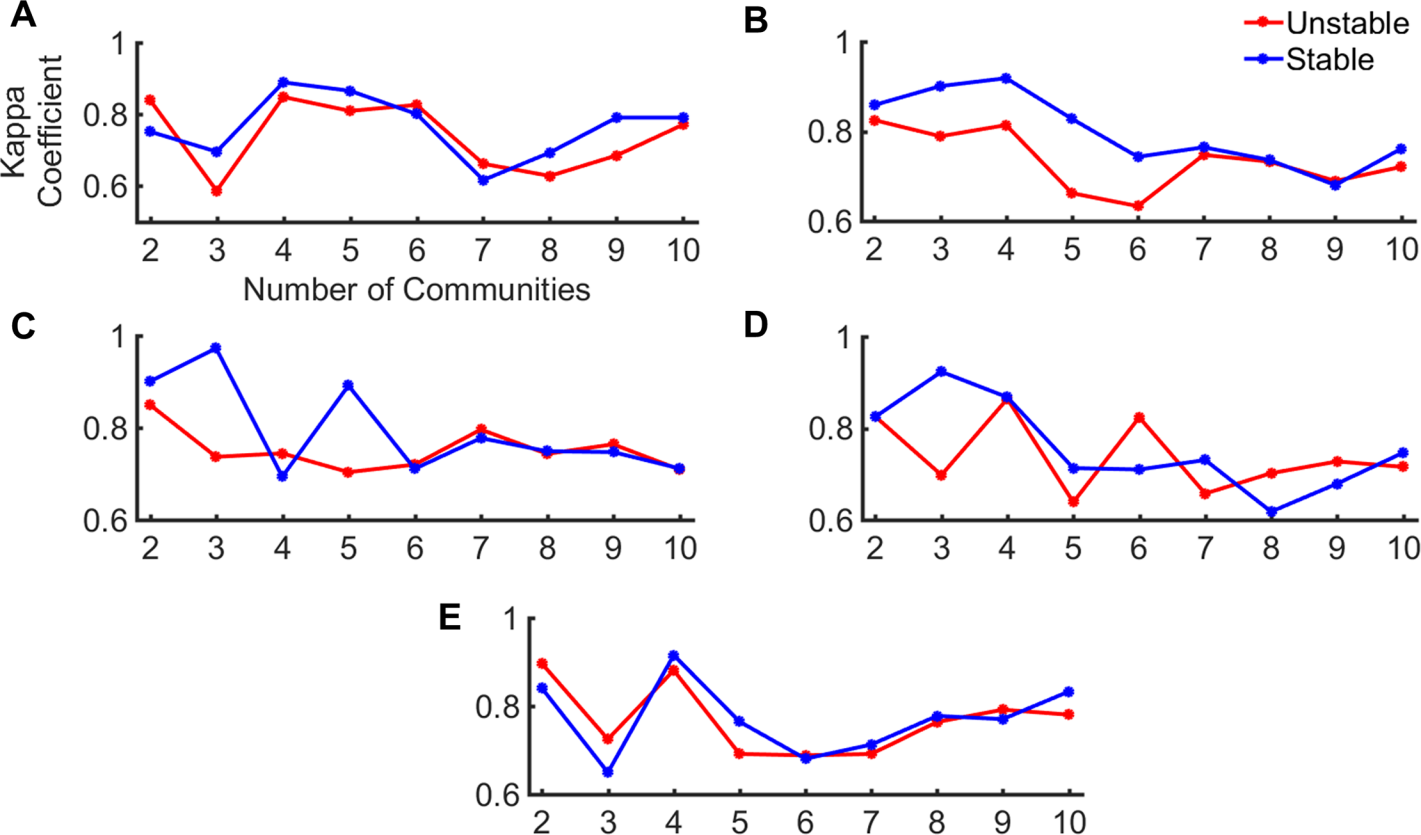
Community agreement for *unstable* (*red*) and *stable* (*blue*) mutants of T4 lysozyme with the wild-type. The figure shows the median kappa coefficient (agreement with wild-type) at each community level for the *unstable* and *stable* mutants. The communities were obtained with *DCC*_*GNM*_ calculated using (A) 5, (B) 10, (C) 20, (D) 30 and (E) 50 low-frequency modes. The abscissa and ordinates correspond to the number of communities and the Kappa coefficient respectively, as given in 6A.

In Fig 6, we show the variation in kappa coefficient for the two mutant categories. For each category, the plot shows the median kappa for individual community levels. It is seen that the s*table* mutants (blue curve) exhibit better agreement with the wild-type than the *unstable* mutants (red curve). Also, it is interesting to note that these differences are manifested in the first 6 communities. At higher community levels, the two mutant types almost come into agreement. It is also interesting to note that this difference in community architecture is more apparent for a subset of 10 modes. To visualize these differences on the protein structures, we consider 3 pairs of *unstable* and *stable* mutants: (PDB IDs: 3c80, 3c81), (PDB IDs: 3c82, 3c81) and (PDB IDs: 3c82, 3c8s). For each pair, we identify the smallest number of communities for which the change is significant. The *ΔΔG* for each of these mutants can be seen in Table 1.

**Fig 7:**
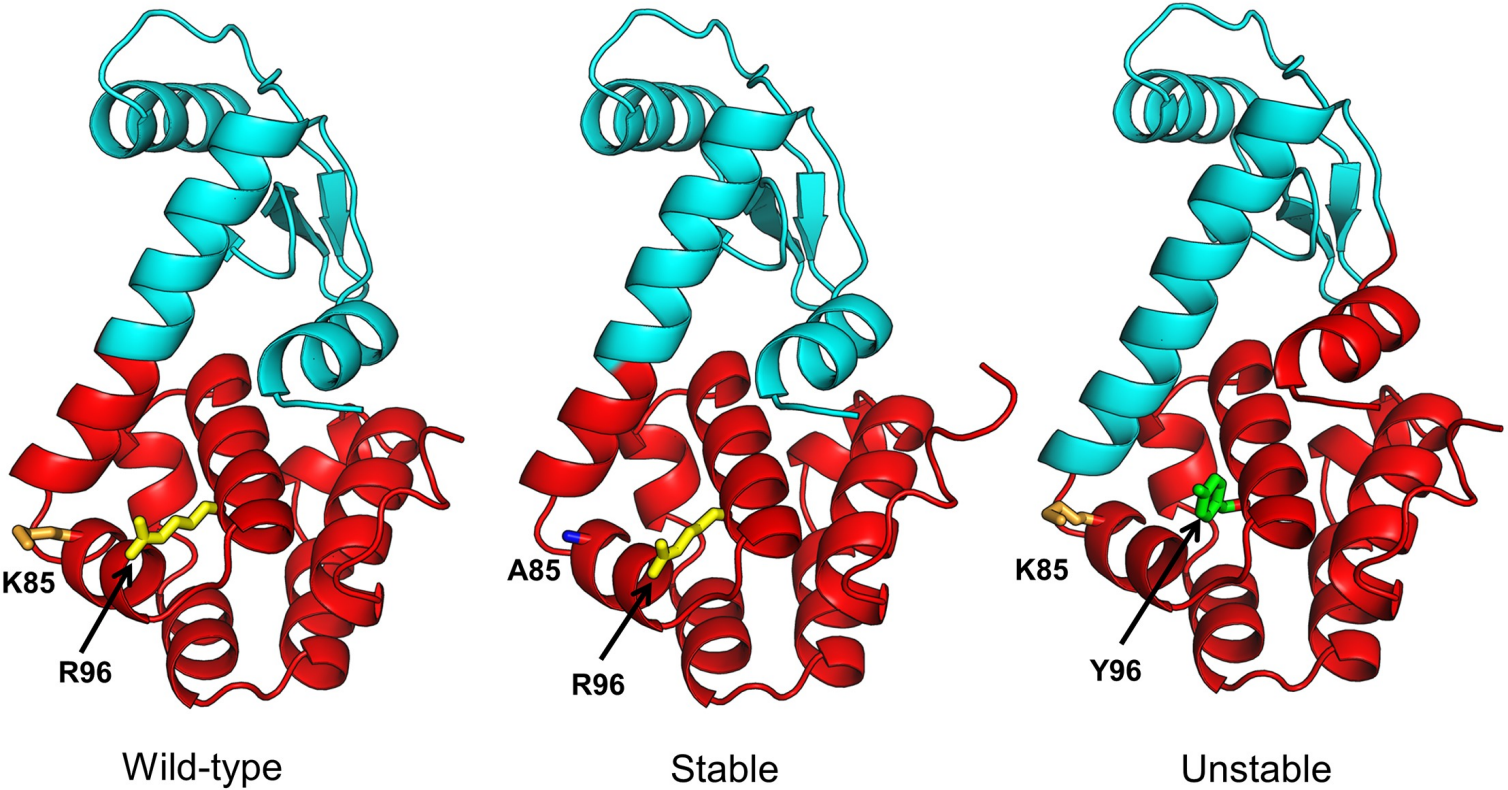
Comparison of community structures for wild-type (PDB: 4s0w), stable (PDB: 3c81) and unstable (PDB: 3c80) mutant forms of T4 lysozyme. Two communities (red and cyan) are shown for each structure. We choose *N*_*c*_ = 2 because the differences in community structure for the stable and unstable forms are most distinctive at this level. Similarly localized communities are colored alike. Sites of mutations are shown in sticks with the corresponding residue names labelled. Side chains of same amino acids in the sites of mutation are colored alike.

**Fig 8:**
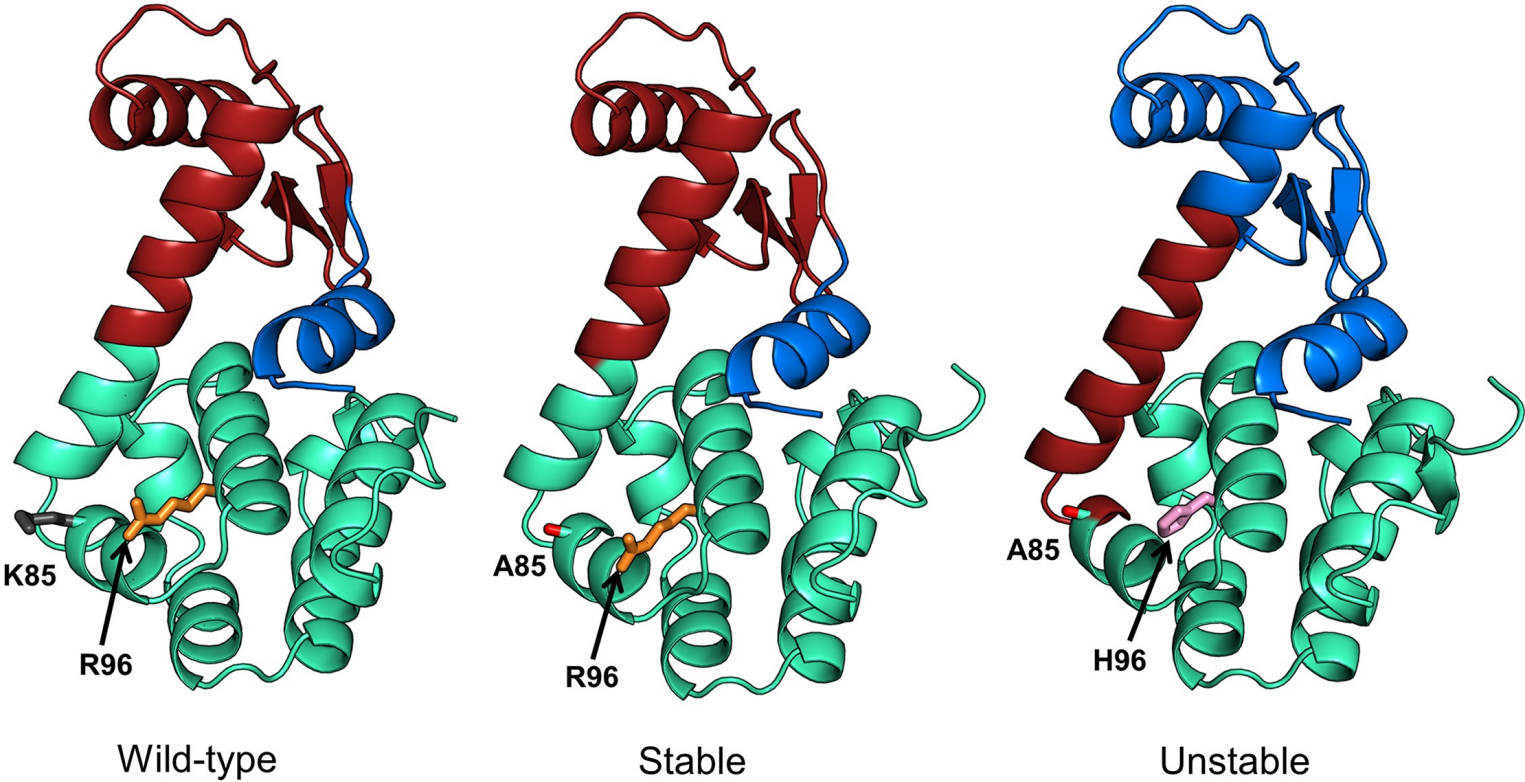
Comparison of community structures for wild-type (PDB: 4s0w), stable (PDB: 3c81) and unstable (PDB: 3c82) mutant forms of T4 lysozyme. Three communities (green, brown and blue) are shown for each structure. *N*_*c*_ = 3 shows maximum structural difference between the community structures of mutant and wild-type forms, hence the choice. Coloring scheme is the same as in Fig 7.

**Fig 9:**
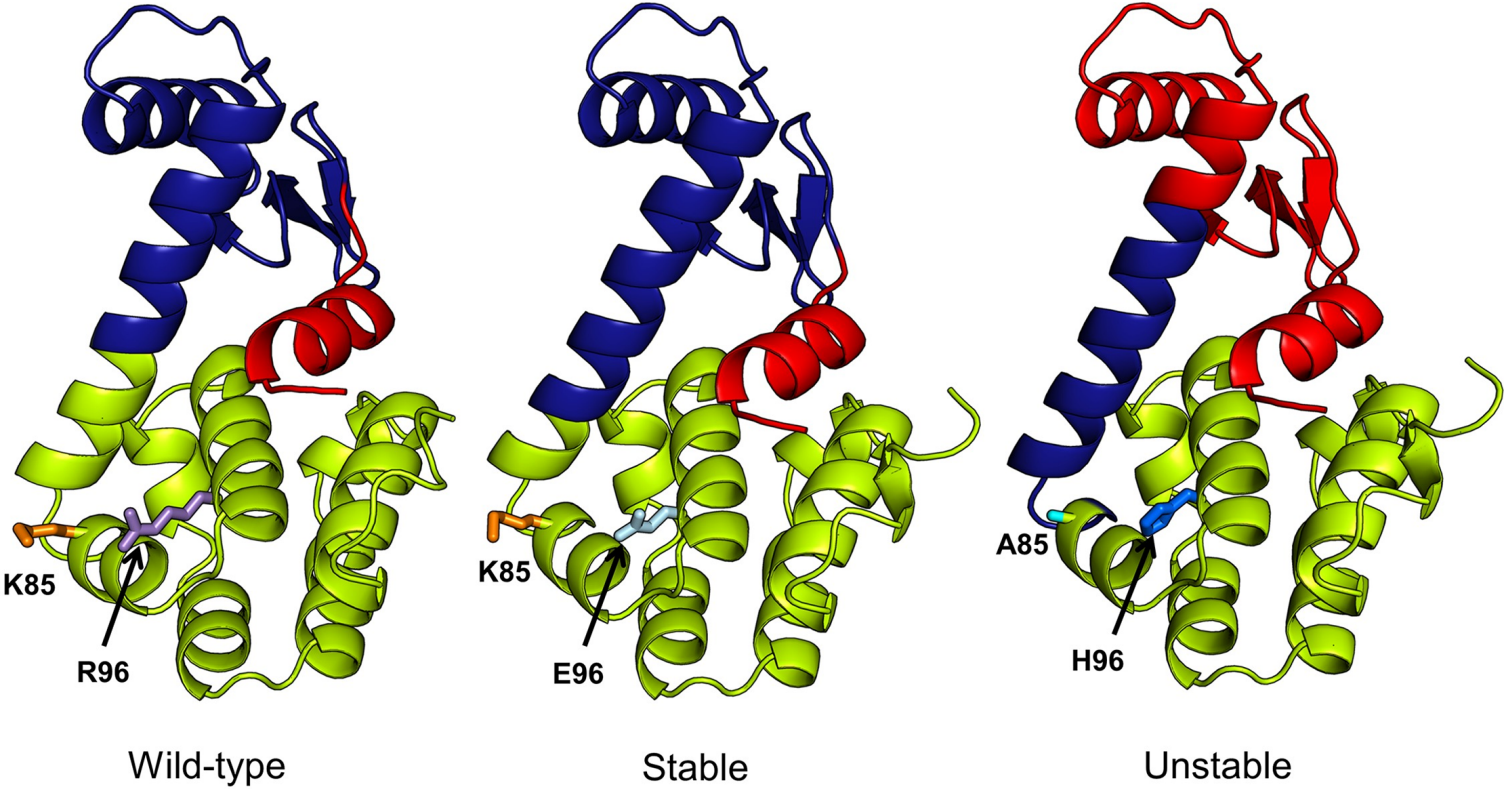
Comparison of community structures for wild-type (PDB: 4s0w), stable (PDB: 3c8s) and unstable (PDB: 3c82) mutant forms of T4 lysozyme. Three communities (red, blue and green) are shown for each structure. *N*_*c*_ = 3 shows the maximum structural differences for the community structures in the mutant and wild-type forms, hence its choice. The coloring scheme is same as in Figs 7 and 8.

Fig 7 (3c80, 3c81), Fig 8 (3c82, 3c81) and Fig 9 (3c82, 3c8s) show communities for each mutant pair relative to the wild-type (4s0w). In each figure, the wild-type structure with the communities is shown on left, the *stable* mutant in the center and the *unstable* mutant on the right. Side chains of mutation sites are shown as sticks with the same residue side chains displayed in the same color. In Fig 7, the difference in community structure for 3c80 (*unstable*) and 3c81 (*stable*) is distinct showing two different communities. The *stable* and *unstable* forms differ visibly in the dynamical correlation of the N-terminal helix (residues 1–12), which is cohesive with the adjacent N-terminal beta sheets and helices in the wild-type and stable forms, while it moves in coordination with the C-terminal domain in the unstable form. The kappa coefficient for the unstable and stable mutant structures is 0.74 and 0.98, respectively. For 3c82 (*unstable*) and 3c81 (*stable*) (Fig 8), the difference is apparent at 3 communities (kappa values of 0.65 and 0.97 respectively). Again we observe a change in the N-terminal helix that moves as an independent unit in the wild-type and *stable* forms, but shows more coordinated motion with the N-terminal domain in the *unstable* form. In Fig 9, we notice the difference at 3 communities and as previously observed, the difference between the *stable* and *unstable* forms becomes visible in the N-terminal helix. The kappa coefficients for the *unstable* (3c82) and *stable* (3c8s) forms at the level of 3 communities are 0.65 and 0.94, respectively.

## Discussion

In the present study, we focus on a simple approach for detecting dynamic communities in proteins with elastic networks. ENM is simpler to formulate, easier to implement and is computationally less expensive in comparison with MD. Here, we emphasize that identifying the true number of dynamic communities is largely an unsolved problem and it is not our current goal to establish ENM as a more accurate method than MD in this aspect. Rather, it is in our interest to show that this method works as well as MD does for community detection. Our results reveal that this single-parameter model can closely reproduce the results from a complex, multi-parameter model like MD, especially for community detection. Owing to its reduced nature, ENM is superior to MD in terms of execution time and thus, can contribute significantly to the investigation of the dynamic communities for larger proteins. We would also like to emphasize that simulation results from MD may not always fully capture the near-native conformation ensemble for a given protein and thus, one should not view results from MD as the absolute truth. The conformational sampling using MD may be highly biased by the simulation length vis a vis the size of the protein, with larger proteins requiring longer simulations to capture a fully representative ensemble of near native conformations. Thus, in our scheme of comparing communities and the underlying correlation matrices obtained from ENM with MD, a lack of agreement between MD and ENM does not necessarily imply the inability of ENM to capture the underlying conformational dynamics. Instead, in some cases, this could be related to the underlying sampling inaccuracies arising from MD.

We show that communities extracted using GNM, a simple formulation of ENM, exhibit a considerable similarity to the communities from MD. We choose GNM over its anisotropic counterpart ANM [21] because it is simpler and because previous studies have shown that GNM exhibits better correlations with experimental B-factors than ANM [38]. Moreover, in a preliminary analysis we observe that the communities obtained with GNM show better agreement with MD than does ANM. In Fig 1, the distance transformed *DCC*_*GNM*_ and *DCC*_*MD*_ matrices for two proteins selected randomly from our dataset show considerable agreement for the regions with high correlation in their dynamics. However, it is surprising to notice a better cohesive behavior, in the case of GNM, showing a close connection between inter-residue dynamical correlations and residue spatial proximity. The dispersion of close contacts suggested by the distance matrix is more closely reproduced with *DCC*_*GNM*_ than with *DCC*_*MD*_. This cohesiveness is a hallmark of the elastic network models in general, and is one reason that they can show better agreement with various protein behaviors than MD. It is however to be noted that we use only the first twenty low-frequency modes from GNM to calculate *DCC*_*GNM*_. As we find in other analysis, the agreement between MD and GNM for different metrics mostly converges for the first 20 normal modes, with the addition of more modes not providing much significant gains.

Our approach to identify dynamic communities differs from existing methods that identify dynamic domains [39,40], which, similar to the approach taken by Kundu [14], divide the structure into rigid units (dynamic domains) based on the sign of the residue positional fluctuations given by the low frequency modes. These methods cluster residues with positive fluctuations into a single group and those with negative fluctuations into a separate group by considering each low frequency mode separately and dividing a protein structure primarily into two rigid clusters or dynamic domains. Depending on the mode that was considered, a single domain may be highly cohesive or may have individual entities that are dispersed over an entire protein structure. In contrast, our approach considers the cumulative contributions from more than one mode by calculating a cross-correlation matrix that combines multiple low frequency modes. Transforming such a matrix into a distance correlation matrix and then clustering it hierarchically, divides the structure into the desired number of dynamic communities based on the extent of inter-residue correlation. While the identification of dynamic domains chooses all residues having the same sign in their positional fluctuations and groups them into one cluster, our method could in principle divide these dynamic domains further into sub-modules, i.e., the dynamic communities.

To model the dynamics, we have considered a fixed distance cutoff *r*_*c*_ = 7.5 Å for each protein. However, it might be more realistic to use a different *r*_*c*_ for each protein, since using a generalized distance cutoff sometimes fails to take into account the size and variations in the packing density in different proteins and may not accurately represent the protein dynamics. Previous implementations of ENM have used a range of different *r*_*c*_ and then considered the *r*_*c*_ that best reproduces the experimental B-factors [15,21].

Our results from comparing the communities obtained upon clustering the distance transformed *DCC*_*GNM*_ and *DCC*_*MD*_ matrices hierarchically, suggest that for a certain number of communities *N*_*c*_, MD and GNM show near-perfect agreement. Importantly, we observe convergence in agreement after using the first few low frequency modes. This also corroborates previous studies that showed that the first few low frequency modes are adequate to reproduce the experimentally observed conformational ensemble of proteins [31,41]. Also, in the case of GNM, though the model assumes isotropic, non-directional residue fluctuations not accounting for the directional preferences of residue mobilities, previous studies have suggested that using the first few low-frequency modes nonetheless results in good correlations with experimental B-factors [42]. When verifying the median kappa for all modes with *r*_*c*_ = 7.5 Å (S1 Fig), it is interesting to note that the median kappa for each subset of modes at all community levels is almost the same (≈ 0.41), except for the subset of 30 modes which shows highest median kappa values. While kappa coefficients of 0.41 rules out the possibility of random agreement, at the same time, one must also consider that there could be possible conformational under-sampling depending on the time scale of the MD trajectory that restricts the extent of agreement between MD and GNM.

Near-convergence for a subset of the first-few low-frequency modes (20–30 modes) is also consistent for the correlation of node centralities and RMSIP between MD and GNM. It is interesting to observe the high correlation for node closeness (0.63), further verifying the strong correspondence between the simulation results from the two methods. However, as Fig 1 suggests, *DCC*_*GNM*_ and *DCC*_*MD*_ do not exhibit 100% agreement with each other. They agree to a large extent in the correlations of secondary structure elements and residues in spatial proximity however, they differ in their scale of inter-residue correlations which could possibly explain the lack of perfect correlation for node closeness.

Singular value decomposition of *DCC*_*GNM*_ and *DCC*_*MD*_ helps in capturing the directions of maximum variations for inter-residue correlations through its principal eigenvectors. Upon verifying the overlap of the principal eigenvectors between MD and GNM we observe an RMSIP of 0.82 (for 20 modes) followed by convergence. This confirms that the *DCC*_*GNM*_ and *DCC*_*MD*_ matrices agree to a large extent in terms of the inter-residue fluctuation correlation. It is also interesting to note that when using either a smaller number of modes (5 modes) or too many modes (50 modes) the standard error in RMSIP increases. While using very few modes possibly leads to a loss in information, including more modes in the calculations for *DCC*_*GNM*_ possibly adds to the noise, since the most reliable modes of motion for the elastic network models are those at the lower frequency end. Higher frequency modes describe local residue-level dynamics and are less reliable. Hence, including those modes in the calculation of the correlation matrix can potentially reduce the signal to noise ratio, resulting in observed lower agreement of *DCC*_*GNM*_ with *DCC*_*MD*_.

The ability of GNM to discriminate stable mutants from unstable ones by evaluating community agreement is notable. The extent of change in community structures in unstable mutants is much greater than for stable mutants. We have used the atomic structures of T4 Lysozyme in the GNM as opposed to the coarse-grained version to account for the mutation changes. Interestingly, we observe that changes to community structures are more distinct in the higher community levels (smaller number of communities) as described by Fig 6. One should consider that we have performed this study only for a set of 16 mutant structures of T4 lysozyme, which is really a very small sample. However, we are limited in the availability of experimentally determined mutant structures for a single protein [43,44]. There is some data for the changes in free energy associated with a single point mutation in proteins [45] however, the crystal structures corresponding to these mutants are not usually available. To use this data, previous methods have considered computational approaches to mutate targeted residues in a given protein and then, used the modeled structure as a representative of the mutant form [46]. However, such computational approaches rely upon the potential function used in the modeling tool and hence, the structure of the modeled mutant (especially the sidechain positions of the mutant site and its neighbors) may be biased by the potential function. The data we have used should be more reliable because these are experimentally reported crystal structures.

## Materials and methods

### Dataset

We compile a set of 44 distinct proteins from the MODEL database [29] by considering only those proteins with MD trajectories of 100 ns or above. Each protein has a minimum of 50 residues. For each protein, we downloaded the all-atom trajectory from the database and parsed the all-atom trajectory into a C^α^ trajectory, having only the coordinates for residue C^α^ atoms in each frame.

### Dynamic cross-correlations from MD trajectory

For each protein, we perform calculations for residue-level dynamic cross-correlations on the respective C^α^ trajectory using the *dccm* function in the Bio3D package [47] with the following equation [48,49].

$$DCC_{MD}(i,j)=\frac{<\Delta r_{i}(t).\Delta r_{j}(t){>}_{t}}{\sqrt{<{\left|\left|\Delta r_{i}(t)\right|\right|}^{2}{>}_{t}}\sqrt{<{\left|\left|\Delta r_{j}(t)\right|\right|}^{2}{>}_{t}}}$$ (1)

Here, *r*_*i*_(*t*) and *r*_*j*_(*t*) refer to the coordinates of the *i*th and *j*th atoms as a function of time *t*, *<*^.^*>* indicates the time ensemble average and Δ*r*_*i*_(*t*) = *r*_*i*_(*t*) − (< *r*_*i*_(*t*) >)_*t*_ and Δ*r*_*j*_(*t*) = *r*_*j*_(*t*) − (< *r*_*j*_(*t*) >)_*t*_.

### Dynamic cross-correlations from Gaussian Network Model

We use GNM [15,50], a form of ENM, to calculate the dynamic cross-correlations between residues. In GNM a protein is usually modeled as a coarse-grained system by representing individual residues by their alpha-carbons, but these points can also be atoms, which we use for the computations on the mutant proteins. Residues within a certain distance cutoff (*r*_*c*_) are connected by Hookean springs. GNM assumes the protein crystal structure to be of energetic minimum conformation and doesn’t require the structure to be energy minimized. It also assumes that residue fluctuations about their mean positions are isotropic and follow a Gaussian distribution in their excursions away from the assumed minimum energy structure. The potential for GNM is given as

$$V=\frac{1}{2}\gamma \sum_{i,j}^{n}\Gamma \;[{\left(\Delta R_{i}-\Delta R_{j}\right)}^{2}]$$ (2)

Here, Δ*R*_*i*_ and Δ*R*_*j*_ are the fluctuation vectors for residue *i* and *j* respectively, *γ* is the stiffness of the springs connecting residues *i* and *j*. *Γ* is the Kirchhoff matrix defining node connectivity and is defined as the following.

$$\Gamma =\left\{ \begin{array}{l} -1,\;if\;i\neq j\;and\;R_{ij}\leq r_{c}\\ \;0,\;if\;i\neq j\;and\;R_{ij}>r_{c}\\ -\sum_{j,j\neq i}\Gamma_{ij},\;if\;i=j \end{array}\right.$$ (3)

Here, *R*_*ij*_ is the distance between the alpha carbons of residues *i* and *j* while, *r*_*c*_ is the distance cutoff. Diagonalizing *Γ* yields *N-1* modes with non-zero eigenvalues. Each mode is a vector that describes the residue fluctuations about its mean position while the eigenvalues correspond to the square of the mode frequency and indicate the relative extent of motion of each point. The slow modes or the low-frequency modes describe the most energetically favorable motions of a protein.

The Kirchhoff matrix has a zero determinant and is thus, singular. The pseudo-inverse of this matrix is calculated using the *N-1* or a subset of the *N-1* modes with the following equation.

$$\Gamma^{-1}=\sum_{i=1}^{N-1}{\lambda_{i}}^{-1}V_{i}V_{i}^{T}$$ (4)

*λ*_*i*_ is the eigenvalue of the i*th* mode, *V*_*i*_ is i*th* mode and $V_{i}^{T}$ is the transpose of *V*_*i*_. The inter-residue dynamical correlation between residues *i* and *j* is then calculated as

$$DCC_{GNM}(i,j)=\frac{\Gamma^{-1}(i,j)}{\sqrt{{(\Gamma }^{-1}(i,i)\;\Gamma^{-1}(j,j))}}$$ (5)

In the present study, we first use a range of different values for the distance cutoff *r*_*c*_ (6, 6.5, 7, 7.5 and 8 Å) and then, select *r*_*c*_ = 7.5 Å, which provides high overlap for dynamics captured from GNM with MD. Using this cutoff, we calculate *DCC*_*GNM*_ using 5, 10, 20, 30 and 50 low-frequency modes.

### Dynamic communities from correlation matrix

For each protein in our dataset, we convert the residue-residue dynamical correlation matrices *DCC*_*MD*_ and *DCC*_*GNM*_ into distance correlation matrices as follows

$$dist\_DCC_{MD}=1-DCC_{MD},$$ (6)

$$dist\_DCC_{GNM}=1-DCC_{GNM}$$ (7)

We then perform hierarchical clustering on the distance correlation matrices with weighted pair-group method with arithmetic mean (WPGMA), which takes into consideration the cluster size when calculating the distance between two clusters [51]. Hierarchical clustering yields dendrograms that can be pruned at different levels to give the desired number of clusters. The clusters obtained upon pruning a dendrogram at a certain height correspond to the dynamic communities, i.e., the blocks of residues that are highly cohesive and move like a rigid body. We cut the dendrograms at different levels to obtain between 2 and 10 communities. The hierarchical clustering was performed using the MATLAB *linkage (https://www.mathworks.com/help/stats/linkage.html)* and *cluster (https://www.mathworks.com/help/stats/cluster.html)* modules.

### Comparing community assignment between MD and GNM

We use 3 metrics to assess the agreement between the communities from MD and GNM.

#### 1. Cohen’s kappa coefficient

The Cohen’s kappa or simply, kappa is a statistic that is often used to evaluate the extent of agreement between data collectors or raters in their assignments to the same variables, referred to as inter-rater reliability. Kappa coefficient is considered to be more robust than percent agreement as it also takes into consideration random agreement [28]. Like correlation coefficients, the value of the kappa statistic can range from -1 to 1. A kappa of 0 indicates an agreement by chance while kappa of 1 indicates perfect agreement [28,30]. We calculate the kappa coefficient as follows

$$K=\frac{p_{o}-p_{e}}{1-p_{e}}$$ (8)

Here, *p*_*o*_ is the observed probability of agreement for cluster assignment between MD and GNM while, *p*_*e*_ is the expected probability of agreement.

#### 2. Network centrality

We model each protein as a weighted network in which a node represents a residue and the edge between a pair of nodes is weighted by the distance transformed correlation for the residue pair (Eq 6 and Eq 7). Then, we calculate the node closeness centralities for the networks from MD and GNM. The closeness centrality is the sum of the lengths of the shortest paths to all other nodes from the given node in the graph. We perform all calculations for network centrality using the MatLab *graph (https://www.mathworks.com/help/matlab/ref/graph.html)* and *centrality (https://www.mathworks.com/help/matlab/ref/graph.centrality.html*) modules.

#### 3. Overlap between principal eigen vectors

We perform singular-value decomposition (SVD) on the *DCC*_*MD*_ and *DCC*_*GNM*_ matrices and then evaluate the overlaps between the MD and GNM eigenvector spaces for subsets of vectors having largest eigenvalues using the root-mean square inner product (RMSIP) [52] as

$$RMSIP=\sqrt{\frac{1}{n}(}\sum_{i=1}^{n}\sum_{j=1}^{n}{\left(V_{i}.U_{j}\right)}^{2}$$ (9)

*V* and *U* are the principal eigenvectors obtained from SVD of the *DCC*_*MD*_ and *DCC*_*GNM*_ matrices respectively, while *n* is the number of vectors to be compared. We consider the same number of principal vectors for the two matrices.

### Mutant dataset

We use PDB structures for the T4 lysozyme mutants crystallized by Mooers *et al*. [37]. In their study, the authors performed circular dichroism assays to estimate stability changes upon specific mutations to the enzyme and calculated the free energy change (*ΔΔG*) for the mutants as *ΔG*_*mutant*_ − *ΔG*_*wildtype*_. The authors have defined the more negative *ΔΔG* values to be the unstable mutants. The stability changes were performed at pH 5.35 and 3.05. In our study, we consider the *ΔΔG* values calculated at pH 5.35. Details of the mutant structures used and their free energy changes with respect to the wild-type are given in Table 1.

### Effect of mutation on dynamic communities

We use all-atom GNM to investigate the community change in the mutant structures with respect to the wild-type. For both the mutant and wild-type forms of the enzyme, we retain all heavy atoms in the PDB and use a distance cutoff of 3.5Å to identify interacting spring locations. Using 5, 10, 20, 30 and 50 modes, we initially calculate the inter-residue dynamical correlations and then, perform hierarchical clustering with weighted average linkage to obtain the desired number of clusters. We trim the dendrograms for each structure at specific heights to obtain 2–10 communities and then compute the agreement between the communities for the wild-type and mutant forms with the kappa coefficient.

## Supporting information

S1 Table Dataset of proteins used in the study. The MD trajectories were downloaded from the MOlecular Dynamics Extended Library (MODEL) database. We retained proteins having at least 50 residues with a minimum simulation length of 100 ns. The table is sorted by the number of residues.
(DOCX)

S2 Table Distribution of *Kappa*_*max*_ for the dataset. For each protein, we identified the community level *N*_*c*_ for which we obtained the maximum value for Kappa coefficient. We show the values for *Kappa*_*max*_ for a subset of 5, 10, 20, 30 and 50 low frequency modes.
(DOCX)

S3 Table Distribution of median kappa coefficient over all community levels for different subsets of modes. For each protein, the table shows the median Kappa over all community levels for each subset of modes.
(DOCX)

S4 Table Correlation for node closeness. The table shows the correlation for node closeness between MD and GNM and the median correlations for each mode. The distance cutoff of 7.5 Å was used for GNM.
(DOCX)

S5 Table Distribution of root-mean square inner product (RMSIP) for the dataset. The principal eigenvectors are obtained with singular-value decomposition of the cross-correlation matrices from MD and GNM. They capture the major directions of variations from the matrix. We see a considerably good overlap (median RMSIP 0.81 over all subsets of modes) between the principal eigenvectors from MD and GNM which suggests a close agreement between the two.
(DOCX)

S1 Fig Distribution of kappa coefficient for all community levels. We verified the variation of Kappa coefficient upon choosing the generalized *r*_*c*_ = 7.5 Å for all proteins. The figure shows the median Kappa over all proteins for all community levels for each subset of modes. The differences in median Kappa for individual subsets of modes is not very high, however the decrease for Kappa with 50 modes following the peak at 30 modes is quite remarkable, emphasizing the importance of the first few low-frequency modes for capturing the global functional dynamics of proteins. The error bars indicate standard error for the Kappa coefficient for a given subset of modes.
(DOCX)
