# Supplementary material for: Protein dynamic communities from elastic network models align closely to the communities defined by molecular dynamics
Source: PLoS One. 2018 Jun 20;13(6):e0199225. doi: 10.1371/journal.pone.0199225 (PMC6010283; doi:10.1371/journal.pone.0199225)
Supplement: S1 Table — The MD trajectories were downloaded from the MOlecular Dynamics Extended Library (MODEL) database. We retained proteins having at least 50 residues with a minimum simulation length of 100 ns. The table is sorted by the number of residues. (DOCX) [file pone.0199225.s001.docx]

S1 Table. Dataset of proteins used in the study. The MD trajectories were downloaded from the MOlecular Dynamics Extended Library (MODEL) database. We retained proteins having at least 50 residues with a minimum simulation length of 100 ns. The table is sorted by the number of residues.

| **PDB ID** | **Simulation Program** | **Duration** | **Protein Name** | **Number of residues** |
| --- | --- | --- | --- | --- |
| 2gb1 | Amber 8 | 1000 ns | Protein G | 56 |
| 1bpi | Amber 8 | 100 ns | Bovine Pancreatic Trypsin Inhibitor | 58 |
| 1g6x | Amber 8 | 100 ns | Pancreatic trypsin inhibitor | 58 |
| 1ark | Amber 8 | 108.93 ns | Nebulin | 60 |
| 1i6f | Amber 8 | 100 ns | Neurotoxin V5 | 60 |
| 1fas | Amber 8 | 100 ns | Fasciculin 1 | 61 |
| 3ci2 | Amber 8 | 100 ns | Chymotrypsin inhibitor 2 | 64 |
| 1csp | Amber 8 | 100 ns | Cold Shock protein | 67 |
| 1sdf | Amber 8 | 100 ns | Stromal Cell Derived factor | 67 |
| 1tba | Amber 8 | 134.2 ns | Transcription initiation factor IID | 67 |
| 1fvq | Amber 8 | 100 ns | Copper transporting ATPase | 72 |
| 1jw2 | Amber 8 | 100 ns | Hemolysin Expression modulating protein | 72 |
| 1txa | Amber 8 | 100 ns | Toxin B | 73 |
| 4icb | Amber 8 | 100 ns | Calbindin D9K | 76 |
| 1sro | Amber 8 | 100 ns | PNPase | 76 |
| 1ubq | Amber 8 | 811.5 ns | Ubiquitin | 76 |
| 1pht | Amber 8 | 100 ns | Phosphatidylinositol kinase | 83 |
| 1cei | Amber 8 | 107.06 ns | Colicin E7 Immunity Protein | 85 |
| 1ls9 | Amber 8 | 100 ns | Cytochrome C6 | 91 |
| 1j5d | Amber 8 | 100 ns | Plastocyanin | 98 |
| 1opc | Amber 8 | 586.22 ns | OMPR | 99 |
| 1kte | Amber 8 | 1001.0 ns | Thioltransferase | 105 |
| 1fkb | Amber 8 | 100 ns | Fk506 Binding Protein | 107 |
| 1nso | Amber 8 | 100.22 ns | Retroviral Protease | 107 |
| 1jli | Amber 8 | 100 ns | Interleukin 3 | 112 |
| 1ooi | Amber 8 | 100 ns | Odorant binding protein (LUSH) | 124 |
| 1agi | Amber 8 | 100 ns | Angiogenin | 125 |
| 1k40 | Amber 8 | 100.22 ns | Adhesin kinase | 126 |
| 1bfg | Amber 8 | 105.832 ns | Basic fibroblast growth factor | 126 |
| 1chn | Amber 8 | 100 ns | CHEY | 126 |
| 1idr | Amber 8 | 159 ns | Hemoglobin Hbn | 126 |
| 1lys | Amber 8 | 329.5 ns | Hen Egg White Lysozyme | 129 |
| 1pdo | Amber 8 | 100 ns | Mannose Permease | 129 |
| 1lit | Amber 8 | 100 ns | Lithostathine | 131 |
| 1cbs | Amber 8 | 100 ns | Cellular retinoic acid binding protein | 137 |
| 1kxa | Amber 8 | 100 ns | Sindbis virus capsid protein | 158 |
| 1emr | Amber 8 | 100 ns | Leukemia Inhibitory Factor | 159 |
| 1czt | Amber 8 | 100 ns | Protein (Coagulation Factor V) | 160 |
| 1il6 | Amber 8 | 100 ns | Interleukin 6 | 166 |
| 1sur | Amber 8 | 100 ns | PAPS Reductase | 215 |
| 1acb | Amber 8 | 100 ns | Alpha Chymotrypsin | 241 |
| 1cgi | Amber 8 | 100 ns | Alpha Chymotrypsinogen | 245 |
| 2hvm | Amber 8 | 100 ns | Hevamine | 273 |
| 1gnd | Amber 8 | 100 ns | Guanine nucleotide dissociation inhibitor | 430 |
