# Supplementary material for: Protein dynamic communities from elastic network models align closely to the communities defined by molecular dynamics
Source: PLoS One. 2018 Jun 20;13(6):e0199225. doi: 10.1371/journal.pone.0199225 (PMC6010283; doi:10.1371/journal.pone.0199225)
Supplement: S2 Table — For each protein, we identified the community level Nc for which we obtained the maximum value for Kappa coefficient. We show the values for Kappamax for a subset of 5, 10, 20, 30 and 50 low frequency modes. (DOCX) [file pone.0199225.s002.docx]

**S2 Table. Distribution of** $\boldsymbol{Kapp}\boldsymbol{a}_{\boldsymbol{max}}$ **for the dataset.** For each protein, we identified the community level $N_{c}$ for which we obtained the maximum value for Kappa coefficient. We show the values for $Kappa_{max}$ for a subset of 5, 10, 20, 30 and 50 low frequency modes.

| **5 modes** | | | **10 modes** | | **20 modes** | | **30 modes** | | **50 modes** | |
| --- | --- | --- | --- | --- | --- | --- | --- | --- | --- | --- |
| **PDB ID** | $\boldsymbol{Kapp}\boldsymbol{a}_{\boldsymbol{max}}$ | $\boldsymbol{N}_{\boldsymbol{c}}$ | $\boldsymbol{Kapp}\boldsymbol{a}_{\boldsymbol{max}}$ | $\boldsymbol{N}_{\boldsymbol{c}}$ | $\boldsymbol{Kapp}\boldsymbol{a}_{\boldsymbol{max}}$ | $\boldsymbol{N}_{\boldsymbol{c}}$ | $\boldsymbol{Kapp}\boldsymbol{a}_{\boldsymbol{max}}$ | $\boldsymbol{N}_{\boldsymbol{c}}$ | $\boldsymbol{Kapp}\boldsymbol{a}_{\boldsymbol{max}}$ | $\boldsymbol{N}_{\boldsymbol{c}}$ |
| 1acb | 0.452 | 9 | 0.371 | 10 | 0.525 | 5 | 0.540 | 3 | 0.576 | 6 |
| 1agi | 0.600 | 3 | 0.625 | 3 | 0.687 | 3 | 0.687 | 3 | 0.599 | 3 |
| 1ark | 0.743 | 4 | 0.682 | 5 | 0.717 | 4 | 0.695 | 4 | 0.723 | 5 |
| 1bfg | 0.450 | 10 | 0.467 | 10 | 0.485 | 9 | 0.492 | 9 | 0.471 | 8 |
| 1bpi | 0.502 | 9 | 0.561 | 2 | 0.653 | 2 | 0.594 | 2 | 0.477 | 10 |
| 1cbs | 0.590 | 2 | 0.484 | 10 | 0.591 | 5 | 0.495 | 8 | 0.651 | 4 |
| 1cei | 0.508 | 8 | 0.477 | 10 | 0.461 | 9 | 0.440 | 10 | 0.429 | 9 |
| 1cgi | 0.434 | 7 | 0.466 | 10 | 0.485 | 9 | 0.565 | 6 | 0.460 | 5 |
| 1chn | 0.545 | 10 | 0.526 | 10 | 0.548 | 10 | 0.459 | 10 | 0.558 | 10 |
| 1csp | 0.541 | 9 | 0.527 | 9 | 0.534 | 2 | 0.607 | 2 | 0.457 | 2 |
| 1czt | 0.265 | 8 | 0.337 | 7 | 0.353 | 9 | 0.323 | 8 | 0.398 | 8 |
| 1emr | 0.437 | 10 | 0.613 | 2 | 0.548 | 2 | 0.460 | 2 | 0.548 | 2 |
| 1fas | 0.413 | 10 | 0.416 | 8 | 0.425 | 8 | 0.448 | 8 | 0.473 | 9 |
| 1fkb | 0.544 | 8 | 0.577 | 9 | 0.619 | 9 | 0.559 | 10 | 0.583 | 10 |
| 1fvq | 0.570 | 8 | 0.749 | 2 | 0.555 | 8 | 0.542 | 10 | 0.649 | 8 |
| 1g6x | 0.466 | 5 | 0.481 | 10 | 0.499 | 10 | 0.528 | 6 | 0.515 | 10 |
| 1gnd | 0.584 | 2 | 0.630 | 2 | 0.833 | 2 | 0.808 | 2 | 0.669 | 2 |
| 1i6f | 0.616 | 6 | 0.489 | 10 | 0.534 | 3 | 0.620 | 6 | 0.559 | 6 |
| 1idr | 0.552 | 7 | 0.585 | 8 | 0.598 | 8 | 0.550 | 9 | 0.608 | 8 |
| 1il6 | 0.488 | 10 | 0.487 | 10 | 0.498 | 7 | 0.496 | 7 | 0.490 | 10 |
| 1j5d | 0.466 | 10 | 0.544 | 9 | 0.495 | 5 | 0.546 | 5 | 0.477 | 8 |
| 1jli | 0.509 | 10 | 0.442 | 9 | 0.581 | 10 | 0.562 | 10 | 0.545 | 10 |
| 1jw2 | 0.483 | 10 | 0.514 | 8 | 0.505 | 9 | 0.567 | 8 | 0.442 | 10 |
| 1k40 | 0.546 | 4 | 0.489 | 4 | 0.614 | 4 | 0.539 | 8 | 0.651 | 3 |
| 1kte | 0.442 | 3 | 0.578 | 5 | 0.466 | 10 | 0.677 | 4 | 0.593 | 8 |
| 1kxa | 0.404 | 9 | 0.402 | 4 | 0.354 | 10 | 0.362 | 9 | 0.381 | 10 |
| 1lit | 0.574 | 9 | 0.584 | 7 | 0.456 | 10 | 0.552 | 9 | 0.558 | 7 |
| 1ls9 | 0.471 | 10 | 0.480 | 9 | 0.480 | 9 | 0.497 | 10 | 0.474 | 8 |
| 1lys | 0.401 | 9 | 0.436 | 10 | 0.445 | 10 | 0.451 | 10 | 0.430 | 10 |
| 1nso | 0.718 | 2 | 0.718 | 2 | 0.718 | 2 | 0.718 | 2 | 0.718 | 2 |
| 1ooi | 0.442 | 9 | 0.428 | 9 | 0.517 | 9 | 0.466 | 9 | 0.489 | 6 |
| 1opc | 0.431 | 5 | 0.488 | 7 | 0.472 | 8 | 0.475 | 5 | 0.483 | 3 |
| 1pdo | 0.466 | 6 | 0.443 | 10 | 0.416 | 6 | 0.521 | 7 | 0.423 | 7 |
| 1pht | 0.635 | 8 | 0.546 | 7 | 0.564 | 7 | 0.589 | 7 | 0.598 | 7 |
| 1sdf | 0.742 | 2 | 0.742 | 2 | 0.742 | 2 | 0.742 | 2 | 0.742 | 2 |
| 1sro | 0.709 | 2 | 0.734 | 2 | 0.502 | 2 | 0.661 | 10 | 0.511 | 9 |
| 1sur | 0.640 | 2 | 0.462 | 10 | 0.434 | 2 | 0.542 | 2 | 0.433 | 5 |
| 1tba | 0.814 | 2 | 0.653 | 4 | 0.640 | 7 | 0.637 | 9 | 0.687 | 9 |
| 1txa | 0.514 | 7 | 0.558 | 8 | 0.531 | 7 | 0.518 | 6 | 0.487 | 7 |
| 1ubq | 0.584 | 7 | 0.626 | 9 | 0.772 | 2 | 0.699 | 2 | 0.539 | 9 |
| 2gb1 | 0.532 | 9 | 0.533 | 9 | 0.626 | 7 | 0.515 | 7 | 0.570 | 8 |
| 2hvm | 0.432 | 8 | 0.439 | 9 | 0.381 | 10 | 0.396 | 9 | 0.442 | 8 |
| 3ci2 | 0.497 | 7 | 0.528 | 10 | 0.493 | 10 | 0.594 | 10 | 0.627 | 7 |
| 4icb | 0.614 | 9 | 0.544 | 3 | 0.626 | 7 | 0.533 | 10 | 0.525 | 9 |
