# Supplementary material for: Protein dynamic communities from elastic network models align closely to the communities defined by molecular dynamics
Source: PLoS One. 2018 Jun 20;13(6):e0199225. doi: 10.1371/journal.pone.0199225 (PMC6010283; doi:10.1371/journal.pone.0199225)
Supplement: S3 Table — For each protein, the table shows the median Kappa over all community levels for each subset of modes. (DOCX) [file pone.0199225.s003.docx]

S3 Table. Distribution of median Kappa coefficient over all community levels for different subsets of modes. For each protein, the table shows the median Kappa over all community levels for each subset of modes.

| **PDB ID** | **Median Kappa**  **(5 modes)** | **Median Kappa**  **(10 modes)** | **Median Kappa**  **(20 modes)** | **Median Kappa**  **(30 modes)** | **Median Kappa**  **(50 modes)** |
| --- | --- | --- | --- | --- | --- |
| 1acb | 0.432 | 0.329 | 0.404 | 0.461 | 0.463 |
| 1agi | 0.494 | 0.536 | 0.503 | 0.469 | 0.511 |
| 1ark | 0.588 | 0.591 | 0.599 | 0.529 | 0.581 |
| 1bfg | 0.290 | 0.343 | 0.341 | 0.351 | 0.340 |
| 1bpi | 0.459 | 0.405 | 0.468 | 0.432 | 0.409 |
| 1cbs | 0.390 | 0.436 | 0.425 | 0.411 | 0.386 |
| 1cei | 0.436 | 0.250 | 0.268 | 0.303 | 0.250 |
| 1cgi | 0.413 | 0.378 | 0.432 | 0.511 | 0.392 |
| 1chn | 0.418 | 0.393 | 0.453 | 0.424 | 0.441 |
| 1csp | 0.437 | 0.501 | 0.421 | 0.436 | 0.371 |
| 1czt | 0.192 | 0.240 | 0.246 | 0.232 | 0.251 |
| 1emr | 0.357 | 0.337 | 0.391 | 0.332 | 0.372 |
| 1fas | 0.292 | 0.287 | 0.353 | 0.403 | 0.284 |
| 1fkb | 0.445 | 0.432 | 0.416 | 0.452 | 0.427 |
| 1fvq | 0.417 | 0.516 | 0.417 | 0.450 | 0.546 |
| 1g6x | 0.367 | 0.450 | 0.428 | 0.464 | 0.367 |
| 1gnd | 0.477 | 0.465 | 0.452 | 0.485 | 0.482 |
| 1i6f | 0.501 | 0.447 | 0.472 | 0.460 | 0.490 |
| 1idr | 0.501 | 0.477 | 0.495 | 0.441 | 0.547 |
| 1il6 | 0.390 | 0.392 | 0.388 | 0.392 | 0.372 |
| 1j5d | 0.395 | 0.460 | 0.428 | 0.458 | 0.365 |
| 1jli | 0.282 | 0.321 | 0.356 | 0.348 | 0.346 |
| 1jw2 | 0.418 | 0.444 | 0.414 | 0.451 | 0.384 |
| 1k40 | 0.445 | 0.445 | 0.490 | 0.417 | 0.480 |
| 1kte | 0.389 | 0.406 | 0.401 | 0.517 | 0.472 |
| 1kxa | 0.364 | 0.388 | 0.295 | 0.332 | 0.325 |
| 1lit | 0.350 | 0.412 | 0.354 | 0.368 | 0.366 |
| 1ls9 | 0.400 | 0.394 | 0.392 | 0.340 | 0.391 |
| 1lys | 0.347 | 0.370 | 0.335 | 0.351 | 0.291 |
| 1nso | 0.363 | 0.369 | 0.368 | 0.348 | 0.349 |
| 1ooi | 0.370 | 0.368 | 0.404 | 0.407 | 0.403 |
| 1opc | 0.391 | 0.404 | 0.426 | 0.447 | 0.429 |
| 1pdo | 0.364 | 0.308 | 0.399 | 0.455 | 0.389 |
| 1pht | 0.537 | 0.484 | 0.464 | 0.490 | 0.464 |
| 1sdf | 0.550 | 0.525 | 0.541 | 0.540 | 0.541 |
| 1sro | 0.519 | 0.563 | 0.412 | 0.527 | 0.408 |
| 1sur | 0.392 | 0.354 | 0.358 | 0.368 | 0.381 |
| 1tba | 0.626 | 0.563 | 0.547 | 0.553 | 0.617 |
| 1txa | 0.475 | 0.473 | 0.481 | 0.422 | 0.406 |
| 1ubq | 0.541 | 0.507 | 0.575 | 0.556 | 0.425 |
| 2gb1 | 0.383 | 0.457 | 0.478 | 0.446 | 0.516 |
| 2hvm | 0.363 | 0.363 | 0.317 | 0.253 | 0.296 |
| 3ci2 | 0.367 | 0.331 | 0.350 | 0.344 | 0.551 |
| 4icb | 0.499 | 0.480 | 0.490 | 0.410 | 0.397 |
