# Supplementary material for: Protein dynamic communities from elastic network models align closely to the communities defined by molecular dynamics
Source: PLoS One. 2018 Jun 20;13(6):e0199225. doi: 10.1371/journal.pone.0199225 (PMC6010283; doi:10.1371/journal.pone.0199225)
Supplement: S4 Table — The table shows the correlation for node closeness between MD and GNM and the median correlations for each mode. The distance cutoff of 7.5 Å was used for GNM. (DOCX) [file pone.0199225.s004.docx]

S4 Table. Correlation for node closeness. The table shows the correlation for node closeness between MD and GNM and the median correlations for each mode. The distance cutoff of 7.5 Å was used for GNM.

| **PDB** | **5 modes** | **10 modes** | **20 modes** | **30 modes** | **50 modes** |
| --- | --- | --- | --- | --- | --- |
| 1acb | 0.101 | -0.045 | 0.533 | 0.558 | 0.639 |
| 1agi | 0.525 | 0.637 | 0.593 | 0.611 | 0.618 |
| 1ark | 0.201 | 0.574 | 0.647 | 0.678 | 0.687 |
| 1bfg | -0.137 | 0.209 | 0.394 | 0.470 | 0.437 |
| 1bpi | 0.260 | 0.640 | 0.661 | 0.765 | 0.812 |
| 1cbs | 0.085 | 0.475 | 0.714 | 0.694 | 0.679 |
| 1cei | 0.245 | 0.276 | 0.118 | 0.084 | 0.164 |
| 1cgi | 0.306 | 0.200 | 0.408 | 0.518 | 0.568 |
| 1chn | 0.011 | 0.617 | 0.683 | 0.674 | 0.650 |
| 1csp | 0.367 | 0.658 | 0.786 | 0.716 | 0.724 |
| 1czt | 0.336 | 0.594 | 0.656 | 0.621 | 0.580 |
| 1emr | -0.117 | 0.300 | 0.478 | 0.538 | 0.667 |
| 1fas | 0.096 | 0.708 | 0.813 | 0.782 | 0.707 |
| 1fkb | -0.169 | 0.611 | 0.769 | 0.757 | 0.745 |
| 1fvq | 0.557 | 0.393 | 0.455 | 0.498 | 0.663 |
| 1g6x | 0.185 | 0.796 | 0.758 | 0.761 | 0.750 |
| 1gnd | 0.438 | 0.559 | 0.564 | 0.638 | 0.674 |
| 1i6f | 0.428 | 0.642 | 0.745 | 0.705 | 0.662 |
| 1idr | 0.192 | 0.449 | 0.585 | 0.467 | 0.436 |
| 1il6 | -0.548 | -0.158 | 0.246 | 0.617 | 0.604 |
| 1j5d | 0.197 | 0.270 | 0.506 | 0.474 | 0.460 |
| 1jli | 0.324 | 0.604 | 0.723 | 0.799 | 0.805 |
| 1jw2 | 0.408 | 0.565 | 0.683 | 0.638 | 0.586 |
| 1k40 | -0.369 | -0.298 | 0.042 | 0.340 | 0.777 |
| 1kte | -0.095 | 0.188 | 0.440 | 0.415 | 0.340 |
| 1kxa | -0.053 | 0.219 | 0.378 | 0.500 | 0.559 |
| 1lit | 0.255 | 0.200 | 0.571 | 0.728 | 0.687 |
| 1ls9 | -0.103 | 0.524 | 0.664 | 0.640 | 0.617 |
| 1lys | 0.286 | 0.560 | 0.610 | 0.700 | 0.513 |
| 1nso | 0.468 | 0.534 | 0.671 | 0.713 | 0.736 |
| 1ooi | 0.059 | -0.233 | 0.371 | 0.510 | 0.430 |
| 1opc | 0.293 | 0.554 | 0.645 | 0.657 | 0.636 |
| 1pdo | 0.038 | 0.472 | 0.614 | 0.582 | 0.533 |
| 1pht | 0.251 | 0.359 | 0.706 | 0.668 | 0.701 |
| 1sdf | 0.778 | 0.922 | 0.859 | 0.894 | 0.899 |
| 1sro | 0.489 | 0.686 | 0.843 | 0.896 | 0.877 |
| 1sur | 0.138 | 0.254 | 0.542 | 0.410 | 0.459 |
| 1tba | 0.441 | 0.450 | 0.474 | 0.454 | 0.631 |
| 1txa | 0.177 | 0.564 | 0.536 | 0.571 | 0.440 |
| 1ubq | -0.423 | -0.256 | 0.035 | 0.396 | 0.161 |
| 2gb1 | 0.038 | 0.310 | 0.357 | 0.363 | 0.229 |
| 2hvm | 0.400 | 0.213 | 0.480 | 0.625 | 0.589 |
| 3ci2 | -0.079 | 0.441 | 0.337 | 0.396 | 0.444 |
| 4icb | 0.624 | 0.820 | 0.665 | 0.700 | 0.811 |
| Median | 0.199 | 0.473 | 0.589 | 0.623 | 0.633 |
