# Supplementary material for: Protein dynamic communities from elastic network models align closely to the communities defined by molecular dynamics
Source: PLoS One. 2018 Jun 20;13(6):e0199225. doi: 10.1371/journal.pone.0199225 (PMC6010283; doi:10.1371/journal.pone.0199225)
Supplement: S5 Table — The principal eigenvectors are obtained with singular-value decomposition of the cross-correlation matrices from MD and GNM. They capture the major directions of variations from the matrix. We see a considerably good overlap (median RMSIP 0.81 over all subsets of modes) between the principal eigenvectors from MD and GNM which suggests a close agreement between the two. (DOCX) [file pone.0199225.s005.docx]

S5 Table. Distribution of root-mean square inner product (RMSIP) for the dataset. The principal eigenvectors are obtained with singular-value decomposition of the cross-correlation matrices from MD and GNM. They capture the major directions of variations. We see a good overlap (median RMSIP 0.81 over all subsets of modes) between the principal eigenvectors from MD and GNM which suggests a similarity between the two dynamics models.

| **PDB** | **5 modes** | **10 modes** | **20 modes** | **30 modes** | **50 modes** |
| --- | --- | --- | --- | --- | --- |
| 1acb | 0.591 | 0.776 | 0.827 | 0.857 | 0.837 |
| 1agi | 0.678 | 0.805 | 0.813 | 0.797 | 0.782 |
| 1ark | 0.813 | 0.856 | 0.815 | 0.829 | 0.921 |
| 1bfg | 0.606 | 0.781 | 0.836 | 0.812 | 0.794 |
| 1bpi | 0.779 | 0.846 | 0.787 | 0.808 | 0.933 |
| 1cbs | 0.704 | 0.790 | 0.852 | 0.835 | 0.793 |
| 1cei | 0.681 | 0.805 | 0.811 | 0.767 | 0.814 |
| 1cgi | 0.575 | 0.768 | 0.804 | 0.841 | 0.830 |
| 1chn | 0.731 | 0.877 | 0.862 | 0.844 | 0.814 |
| 1csp | 0.771 | 0.802 | 0.817 | 0.806 | 0.887 |
| 1czt | 0.673 | 0.786 | 0.821 | 0.830 | 0.799 |
| 1emr | 0.617 | 0.780 | 0.832 | 0.837 | 0.784 |
| 1fas | 0.801 | 0.843 | 0.810 | 0.819 | 0.923 |
| 1fkb | 0.833 | 0.878 | 0.875 | 0.825 | 0.794 |
| 1fvq | 0.701 | 0.824 | 0.793 | 0.779 | 0.859 |
| 1g6x | 0.782 | 0.824 | 0.791 | 0.805 | 0.938 |
| 1gnd | 0.735 | 0.747 | 0.807 | 0.829 | 0.844 |
| 1i6f | 0.748 | 0.815 | 0.794 | 0.817 | 0.922 |
| 1idr | 0.729 | 0.834 | 0.843 | 0.810 | 0.793 |
| 1il6 | 0.660 | 0.755 | 0.854 | 0.852 | 0.786 |
| 1j5d | 0.678 | 0.760 | 0.796 | 0.779 | 0.793 |
| 1jli | 0.635 | 0.800 | 0.833 | 0.812 | 0.794 |
| 1jw2 | 0.785 | 0.807 | 0.809 | 0.786 | 0.836 |
| 1k40 | 0.784 | 0.851 | 0.872 | 0.812 | 0.794 |
| 1kte | 0.695 | 0.812 | 0.826 | 0.802 | 0.785 |
| 1kxa | 0.669 | 0.807 | 0.824 | 0.814 | 0.783 |
| 1lit | 0.726 | 0.810 | 0.869 | 0.838 | 0.820 |
| 1ls9 | 0.686 | 0.842 | 0.810 | 0.786 | 0.824 |
| 1lys | 0.733 | 0.830 | 0.858 | 0.840 | 0.807 |
| 1nso | 0.636 | 0.737 | 0.779 | 0.781 | 0.785 |
| 1ooi | 0.721 | 0.822 | 0.866 | 0.826 | 0.789 |
| 1opc | 0.692 | 0.835 | 0.849 | 0.812 | 0.811 |
| 1pdo | 0.763 | 0.819 | 0.831 | 0.824 | 0.797 |
| 1pht | 0.811 | 0.864 | 0.841 | 0.792 | 0.818 |
| 1sdf | 0.824 | 0.855 | 0.792 | 0.818 | 0.896 |
| 1sro | 0.717 | 0.813 | 0.813 | 0.796 | 0.857 |
| 1sur | 0.779 | 0.782 | 0.841 | 0.832 | 0.807 |
| 1tba | 0.819 | 0.830 | 0.799 | 0.783 | 0.886 |
| 1txa | 0.669 | 0.791 | 0.806 | 0.798 | 0.853 |
| 1ubq | 0.774 | 0.867 | 0.842 | 0.804 | 0.856 |
| 2gb1 | 0.775 | 0.860 | 0.795 | 0.800 | 0.941 |
| 2hvm | 0.713 | 0.769 | 0.832 | 0.847 | 0.838 |
| 3ci2 | 0.758 | 0.821 | 0.816 | 0.799 | 0.890 |
| 4icb | 0.696 | 0.832 | 0.804 | 0.794 | 0.841 |
| Median | 0.724 | 0.814 | 0.819 | 0.812 | 0.819 |
