# Supplementary material for: Protein dynamic communities from elastic network models align closely to the communities defined by molecular dynamics
Source: PLoS One. 2018 Jun 20;13(6):e0199225. doi: 10.1371/journal.pone.0199225 (PMC6010283; doi:10.1371/journal.pone.0199225)
Supplement: S1 Fig — We verified the variation of Kappa coefficient upon choosing the generalized rc = 7.5 Å for all proteins. The figure shows the median Kappa over all proteins for all community levels for each subset of modes. The differences in median Kappa for individual subsets of modes is not very high, however the decrease for Kappa with 50 modes following the peak at 30 modes is quite remarkable, emphasizing the importance of the first few low-frequency modes for capturing the global functional dynamics of proteins. The error bars indicate standard error for the Kappa coefficient for a given subset of modes. (DOCX) [file pone.0199225.s006.docx]

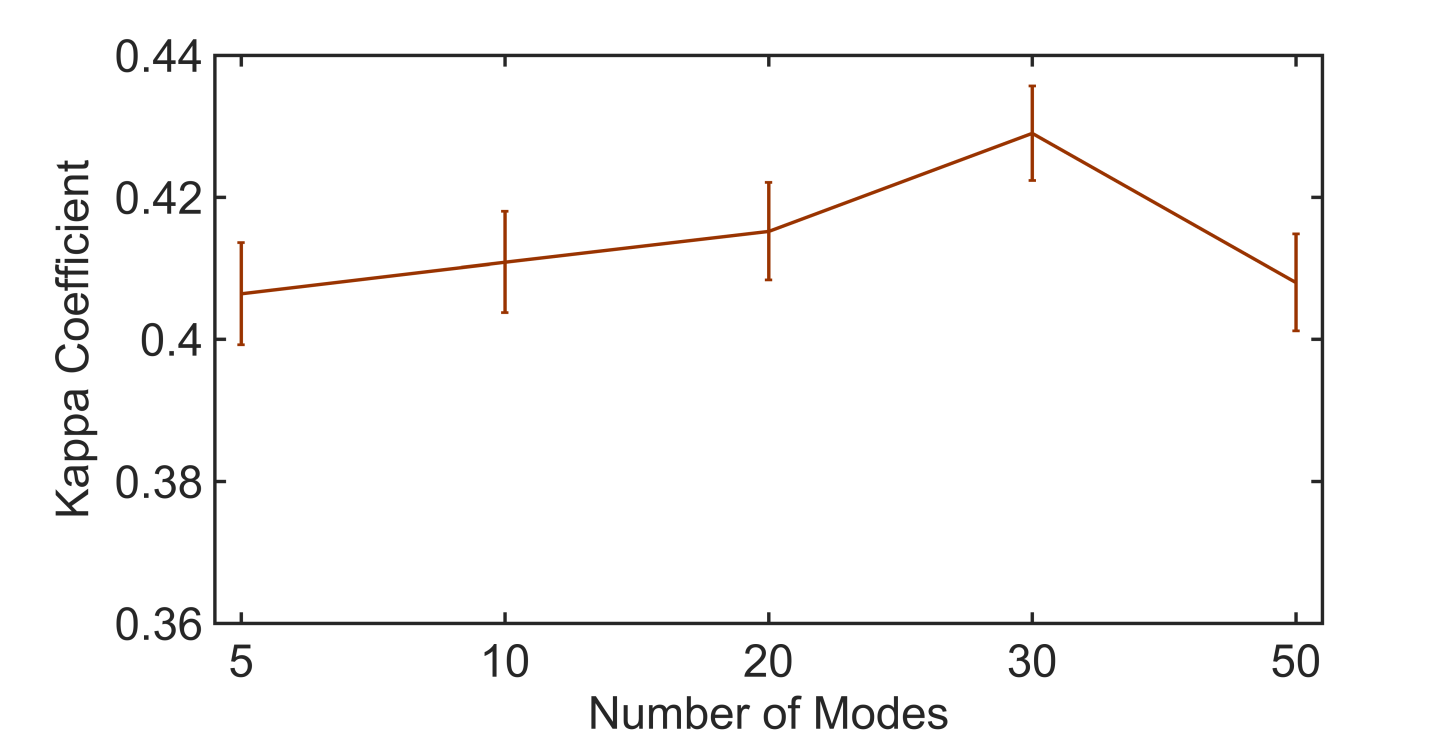


S1 Fig. Distribution of Kappa coefficient for all community levels. We verified the variation of Kappa coefficient upon choosing the $r_{c}$ = 7.5 Å used for all proteins. The figure shows the median Kappa over all proteins for all community levels for each subset of modes. The differences in median Kappa for individual subsets of modes is not very high, however the decrease for Kappa with 50 modes following the peak at 30 modes is somewhat surprising, emphasizing the importance of the first few low-frequency modes for capturing the global functional dynamics of proteins. The error bars indicate standard error for the Kappa coefficient for a given subset of modes.
